# NAD^+^ Metabolism Licenses Zygotic Genome Activation via PARP7‐Mediated ADP‐Ribosylation of UHRF1 in Mouse Early Embryos

**DOI:** 10.1002/advs.76136

**Published:** 2026-06-15

**Authors:** Guangyi Cao, Ziqi Zhang, Anqi Chen, Yanbo Liu, Yuling Lin, Lina Yu, Ruixin Shi, Aolei Guo, Yan Mao, Ganggui Lou, Chaojun Li, Luhong Wen, Guijun Yan, Haixiang Sun

**Affiliations:** ^1^ State Key Laboratory of Reproductive Medicine and Offspring Health Center for Reproductive Medicine and Obstetrics and Gynecology Nanjing Drum Tower Hospital Clinical College of Nanjing Medical University Nanjing China; ^2^ Center for Reproductive Medicine and Obstetrics and Gynecology Nanjing Drum Tower Hospital Affiliated Hospital of Medical School Nanjing University Nanjing China; ^3^ Center for Reproductive Medicine and Obstetrics and Gynecology Joint Institute of Nanjing Drum Tower Hospital for Life and Health College of Life Science Nanjing Normal University Nanjing China; ^4^ Jiangsu Human Reproductive Function Remodeling Engineering Research Center Nanjing China; ^5^ Nanjing Clinical Medical Center for Reproductive Medicine Nanjing Jiangsu China; ^6^ The Research Institute of Advanced Technologies Ningbo University Ningbo China; ^7^ China Innovation Instrument Company Ltd. Ningbo China

**Keywords:** adp‐ribosylation, chromatin accessibility, metabolomics, nad^+^ metabolism, parp7, zygotic genome activation

## Abstract

Zygotic genome activation (ZGA) is a critical developmental milestone whose metabolic regulation remains unclear. This study identifies a pivotal role for Nicotinamide adenine dinucleotide (NAD^+^) metabolism in regulating ZGA through poly(ADP‑ribose) polymerase 7(PARP7)‐mediated ADP‐ribosylation. Using ultra‐low input embryo metabolomics, we profiled metabolism from zygote to blastocyst, revealing a significant NAD^+^ decline at the 2‐cell stage. This shift coincided with specific upregulation of the mono‐ADP‐ribosyltransferase PARP7, confirmed by transcriptomics, quantitative RT‐PCR, western blot, and immunofluorescence. Genetic knockdown via trim‐away technology or pharmacological inhibition with RBN‐2397 caused developmental delay/arrest at the 2‐cell stage, impaired blastocyst formation, and defective ZGA. Mechanistically, PARP7 deficiency reduced chromatin accessibility (ATAC‐seq), diminished H3K4ac and H3K27ac marks, and impaired RNA polymerase II transcription. Integrated proteomics and ADP‐ribosylome analysis of late 2‐cell embryos identified UHRF1 as a key PARP7 target, mono‐ADP‐ribosylated at lysines K30 and K31. This modification stabilized UHRF1 protein (cycloheximide chase), and UHRF1 overexpression partially rescued the transcriptional defects associated with ZGA from PARP7 inhibition. Our findings establish a metabolic‐epigenetic axis wherein NAD^+^ metabolism, via PARP7‐mediated ADP‐ribosylation of UHRF1, regulates chromatin remodeling and transcriptional activation during ZGA, offering fundamental insights into early development.

## Graphical abstract

This study uncovers a metabolic‐epigenetic axis licensing zygotic genome activation (ZGA) in mouse embryos. A developmental decline in NAD^+^ levels activates PARP7, which mono‐ADP‐ribosylates and stabilizes UHRF1. This modification promotes the establishment of permissive histone acetylation marks, thereby facilitating timely ZGA. This work directly links nutrient metabolism with epigenetic reprogramming in early development.

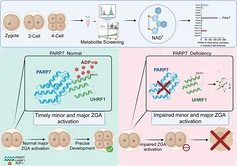

## 1 Introduction

Zygotic genome activation (ZGA) is the foundational transition from maternal to embryonic control, a process absolutely required for preimplantation development. In mice, this involves a precisely timed cascade: a minor activation wave followed by the major transcriptional burst during the mid‐to‐late 2‐cell stage [1, 2]. The execution of ZGA is not merely a function of assembling the core transcriptional machinery; it is equally dependent on extensive chromatin remodeling and the establishment of permissive histone modification landscapes, such as the dynamic redistribution of H3K4me3 and the deposition of H3K27ac at enhancers [3, 4]. Mounting evidence suggests that this epigenetic reprogramming is not autonomous but is intricately coupled to the metabolic state of the embryo, forming a critical metabolic‐epigenetic axis that gates developmental progression [5, 6]. A key, unresolved question is how specific metabolic cues are sensed and transduced into the epigenetic and transcriptional changes that drive ZGA.

Nicotinamide adenine dinucleotide (NAD^+^) sits at a crucial nexus of this regulation. Beyond its canonical role in redox reactions, NAD^+^ serves as an essential substrate for NAD^+^‐consuming enzymes, including sirtuins and ADP‐ribosyltransferases (PARPs), thereby directly linking cellular metabolism to epigenetic and post‐translational modification signaling [7, 8]. While declining NAD^+^ levels are associated with oocyte aging and quality decline [9], the dynamics, regulation, and functional impact of NAD^+^ metabolism during the critical window of preimplantation development, particularly at the 2‐cell stage, remain incompletely understood. This gap in knowledge leaves a fundamental disconnect in our understanding of how metabolic flux influences developmental competence.

Among NAD^+^‐consuming enzymes, the PARP family catalyzes the transfer of ADP‐ribose moieties onto target proteins. Poly(ADP‑ribose) polymerase 7 (PARP7; also known as TIPARP), a mono‐ADP‐ribosyltransferase (MART), is emerging as a key regulator of gene expression and cellular stress responses in somatic contexts, including antiviral immunity and tumorigenesis [10, 11, 12]. Notably, PARP7 modifies transcription‐associated proteins, positioning it as a potential direct mediator between metabolism and transcription [13, 14]. Despite this, its role in the unique context of the early embryo, where rapid metabolic shifts and global transcriptional awakening coincide, is entirely unexplored. Its expression pattern, regulation, and potential function in interpreting metabolic states (like NAD^+^ availability) to orchestrate ZGA represent a significant and compelling biological mystery.

In this study, we hypothesized that dynamic NAD^+^ metabolism provides a critical signal for ZGA through the action of stage‐specific NAD^+^‐consuming enzymes. Using ultra‐low‐input metabolomics, we first mapped the metabolic landscape of preimplantation development and identified a pronounced, stage‐specific depletion of NAD^+^ at the 2‐cell stage. This metabolic shift prompted us to investigate PARP7, which we discovered is specifically upregulated during this critical period. Through a multi‐omics approach integrating functional perturbations (genetic knockdown and pharmacological inhibition with the selective inhibitor RBN‐2397), live imaging, transcriptomics (RNA‐seq), epigenomics (ATAC‐seq, CUT&Tag), and ADP‐ribosylomics, we elucidate a novel pathway. We demonstrate that PARP7 acts as a metabolic sensor, using NAD^+^ to catalyze the mono‐ADP‐ribosylation of the epigenetic regulator UHRF1, thereby stabilizing it to facilitate chromatin opening and the activation of RNA Polymerase II, which is essential for successful ZGA. Our findings define a direct metabolic‐epigenetic circuit where a metabolite (NAD^+^) and its cognate enzyme (PARP7) coordinate to license the embryonic genome for activation.

## 2 Results

### 2.1 Low‐Input Embryonic Metabolomics Reveals a Significant Decrease in NAD^+^ at the 2‐Cell Stage, Which Inversely Correlates With the Expression of the NAD^+^ Hydrolase PARP7

**FIGURE 1:**
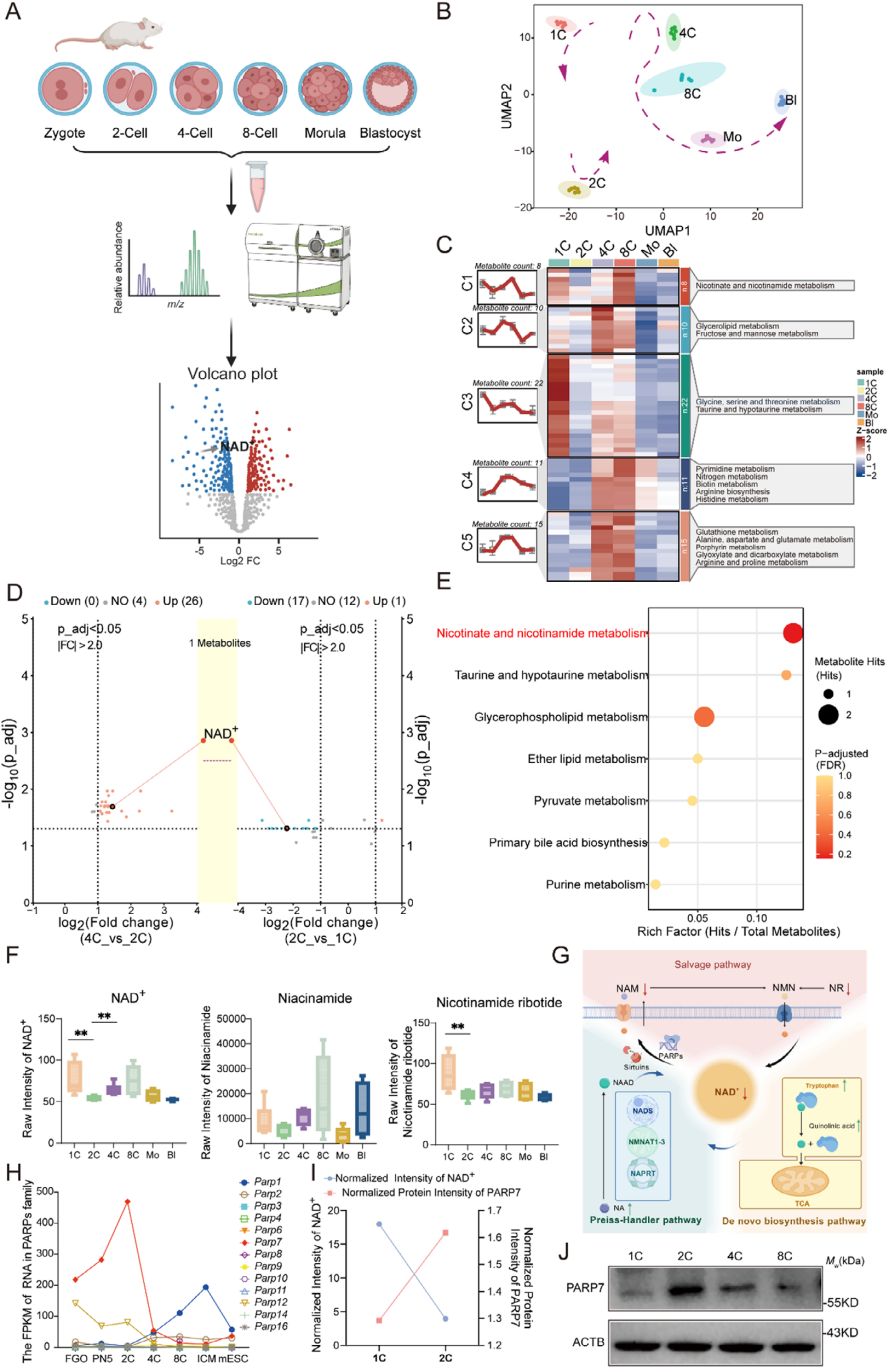
The landscape of micro‐scale embryonic metabolomics reveals a significant decrease in NAD^+^ at the two‐cell stage. (A) Schematic diagram of the metabolomics experimental workflow. Mouse embryos from six developmental stages (from zygote to blastocyst) were collected. Samples were analyzed using a single‐cell mass spectrometer (SinCell‐100) in positive‐ion targeted multiple reaction monitoring (MRM) mode. A custom algorithm was used to extract the absolute intensity matrix based on parent‐daughter ion pairs. (B) UMAP clustering plot of the metabolomics data, showing clear separation among different developmental stages. Zygote, 1C;2‐Cell, 2C;4‐Cell, 4C;8‐Cell, 8C; Morula, Mo; Blastocyst, Bl. (C) Heatmap of K‐means clustered metabolites (Cluster 1–5). Left: Trend of standardized metabolite abundance within each cluster. Right: Representative KEGG pathways enriched in each cluster. Cluster 1 highlights the down‐regulation of nicotinate and nicotinamide metabolism. (D) Volcano plots for 4C vs. 2C (left) and 2C vs. 1C (right). Red/blue dots represent significantly differential metabolites (|log_2_FC| > 1, *p* < 0.05). NAD^+^‐related precursors are labeled. (E) KEGG enrichment analysis based on differential metabolites significantly down‐regulated at the 2‐cell stage (2Cell vs 1Cell), highlighting the “Nicotinate and nicotinamide metabolism” pathway. (F) Box plots showing the raw intensities of NAD^+^, nicotinamide, and nicotinamide riboside across developmental stages. (G) Schematic diagram of NAD^+^ synthesis and salvage pathways. Created with BioGDP.com. (H) FPKM of Parp family genes; Parp7 shows a peak of specific expression at the two‐cell stage. (I) Correlation analysis between normalized NAD^+^ intensity (blue) and normalized PARP7 protein intensity (pink) during the 1‐ to 2‐cell transition, showing a negative correlation. (J) Representative Western blot results of PARP7 protein levels from the zygote to 8‐cell stage, using β‐actin as the internal reference.

To investigate the dynamics of metabolic pathways during early embryonic development, we collected embryos at six developmental stages (zygote, 2‐cell, 4‐cell, 8‐cell, morula, and blastocyst), with each sample comprising 10 embryos and six biological replicates per stage (Figure 1A). All metabolites identified were listed in the source data. UMAP analysis demonstrated that low‐input metabolomics could distinctly separate all six developmental stages (Figure 1B). Unsupervised clustering of the metabolomic data across time points revealed five distinct clusters (C1–C5) with characteristic expression patterns. Metabolites in cluster C1 were highly abundant at the zygote and 8‐cell stages and were primarily enriched in pathways such as “Nicotinate and nicotinamide metabolism.” Cluster C2 metabolites were highly expressed at the 4‐cell and 8‐cell stages, with a further increasing trend in blastocysts, and were enriched in pathways like “Glycerolipid metabolism.” Metabolites in cluster C3 showed high expression at the zygote stage, enriched in pathways such as “Glycine, serine and threonine metabolism.” Cluster C4 metabolites were highly expressed at the 2‐cell, 4‐cell, and 8‐cell stages, associated with pathways like “Pyrimidine metabolism.” Finally, cluster C5 metabolites were highly expressed at the 4‐cell and 8‐cell stages, enriched in pathways including “Glutathione metabolism” (Figure 1C). Volcano plot analysis confirmed significant metabolic differences between each pair of adjacent embryonic stages (Figure S1A). Notably, eight metabolites, including NAD^+^, were significantly downregulated at the 2‐cell stage (2‐cell vs zygote) and subsequently upregulated at the 4‐cell stage (4‐cell vs 2‐cell) (Figure 1D). Gene Ontology (GO) analysis of differentially expressed metabolites between the 2‐cell and zygote stages revealed significant enrichment in pathways such as “Nicotinate and nicotinamide metabolism” (Figure 1E). Targeted metabolomics revealed that NAD^+^ levels were significantly decreased at the 2C stage. Consistently, its precursor nicotinamide riboside was also downregulated. In contrast, the level of nicotinamide remained relatively stable during this transition (Figure 1F). This marked reduction of NAD^+^, the essential co‐substrate for PARP family enzymes, suggests a potential shift in NAD^+^ metabolism coinciding with ZGA. While declining NAD^+^ levels due to excessive consumption (e.g., by PARP activation) or reduced synthesis are known to contribute to metabolic dysfunction and reduced oocyte quality during aging [9, 15] (Figure 1G), the cause and functional significance of this specific, stage‐dependent NAD^+^ depletion during the zygote‐to‐2‐cell transition remain unknown.

The ADP‐ribosyltransferase (PARP) family (PARP1–PARP16) comprises NAD^+^ hydrolases that utilize NAD^+^ as a substrate, hydrolyzing it to ADP‐ribose (ADPR) and nicotinamide (NAM). PARP enzymes catalyze the transfer of the ADP‐ribose moiety from NAD^+^ to target proteins, a reaction termed ADP‐ribosylation [16, 17]. However, the role of PARP family members during early embryonic development remains largely unexplored. We found that PARP7, a member of the PARP family, exhibits specific and marked upregulation at the 2‐cell stage, as revealed by re‐analysis of published transcriptome data (GSE165782) [18] (Figure 1H). Intriguingly, the expression trend of the NAD^+^ hydrolase PARP7 showed an inverse correlation with NAD^+^ levels during the zygote‐to‐2‐cell transition (Figure 1I). Although sirtuin family proteins can also hydrolyze NAD^+^, the protein levels of sirtuins expressed in embryos (SIRT1, SIRT2, SIRT4, SIRT5, SIRT6) did not show a corresponding inverse dynamic at the 2‐cell stage (Figure S1B) [19]. Western blot analysis further confirmed that PARP7 protein is expressed throughout early development and is significantly increased at the 2‐cell stage compared to the zygote stage (Figure 1J; Figure S1C).

The low‐input embryonic metabolomic landscape reveals a significant decrease in NAD^+^ at the 2‐cell stage, which inversely correlates with the expression of the NAD^+^ hydrolase PARP7, suggesting a potential correlation between NAD^+^ and PARP7. However, the reason for and functional consequence of NAD^+^ consumption at the 2‐cell stage remain unclear.

### 2.2 Knockdown of PARP7 Leads to 2‐Cell Developmental Delay and Reduced Blastocyst Rate

**FIGURE 2:**
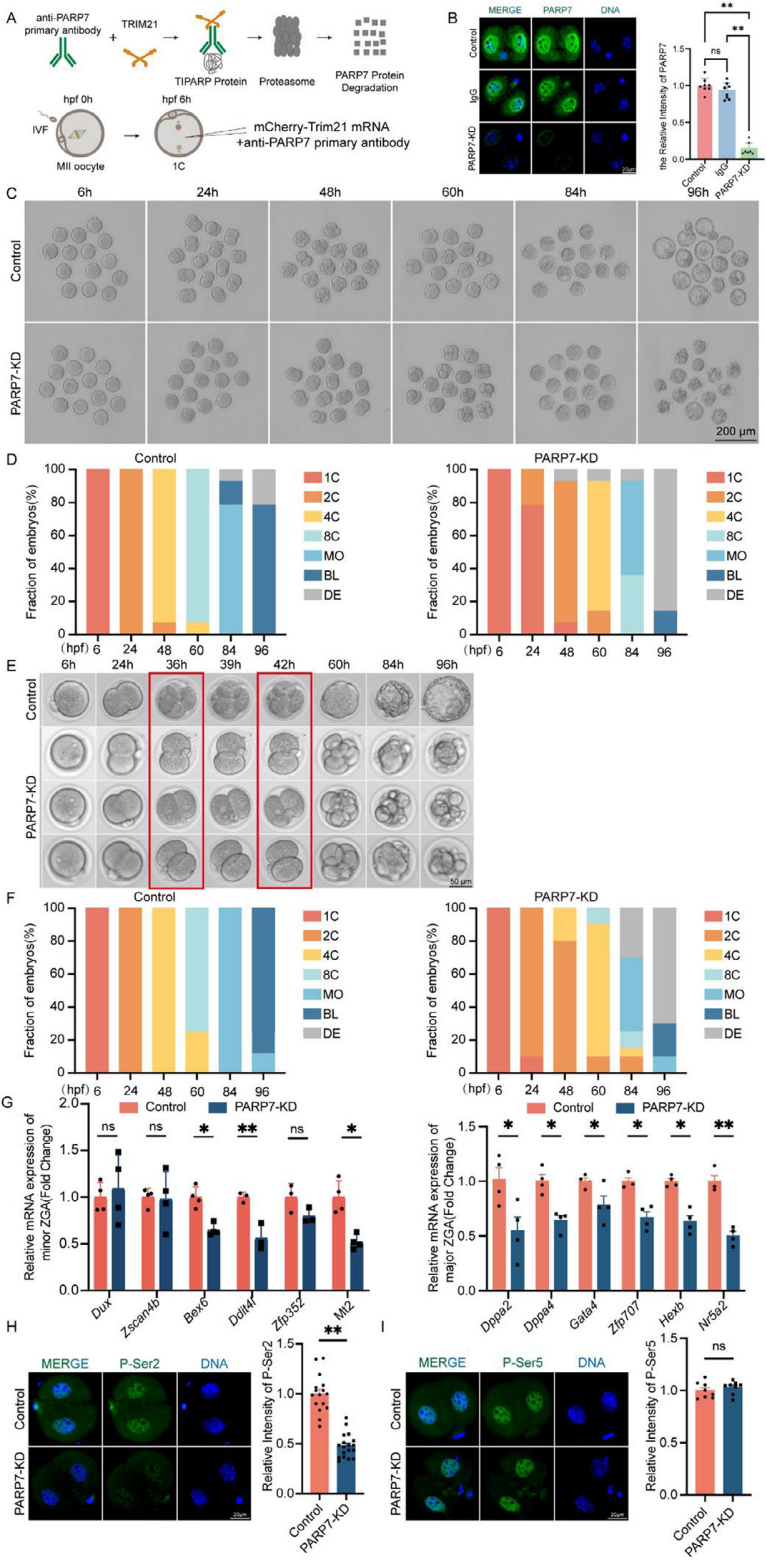
PARP7 knockdown leads to developmental delay at the two‐cell stage and reduces blastocyst rate. (A) Schematic diagram of the Trim‐Away strategy for acute degradation of PARP7. Embryos were microinjected with anti‐PARP7 antibody and mCherry‐Trim21 mRNA at 6 h post‐fertilization (hpf). (B) Representative immunofluorescence images showing efficient degradation of PARP7 protein in 2‐cell stage embryos. PARP7 is green, and DNA was stained with DAPI (blue). Scale bar: 20 µm. (C) Representative bright‐field images of control and PARP7‐KD (knockdown) embryos during culture from 6 to 96 hpf. Scale bar: 200 µm. (D) Embryos at specified developmental stages from control and PARP7‐KD groups were quantified at the indicated time points (N>60 per group from three independent experiments). (E) Representative time‐lapse morphological tracking of embryos. The red box highlights the developmental arrest during the 2‐ to 4‐cell transition in PARP7‐KD embryos compared to controls. Scale bar: 50 µm. (F) Stacked bar chart showing the percentage of embryos at each developmental stage corresponding to the experiment in panel E. (G) RT‐qPCR analysis of Minor ZGA genes (left) and Major ZGA genes (right) in 2‐cell embryos. PARP7 deficiency significantly downregulated the expression of key ZGA markers. (H, I) Representative immunofluorescence images and quantitative analysis of RNA polymerase II C‐terminal domain serine 2 (P‐Ser2) phosphorylation (H) and serine 5 (P‐Ser5) phosphorylation (I) in 2‐cell embryos. PARP7‐KD significantly reduced P‐Ser2 intensity but not P‐Ser5 intensity. Scale bar: 20 µm. Data are presented as mean ± SEM. n = 3 independent biological replicates. Statistical analysis was performed using Student's t‐test. ^*^ *p* < 0.05, ^**^ *p* < 0.01, ns indicates no significant difference.

To investigate the role of PARP7 in early embryonic development, we employed the “trim‐away” technique [20] by co‐injecting zygotes with *Trim21* mRNA and a PARP7 antibody to achieve targeted protein degradation (Figure 2A). Immunofluorescence confirmed a significant reduction of nuclear PARP7 signal in PARP7‐knockdown (PARP7‐KD) 2‐cell embryos, with the intensity decreased by approximately 80% (Figure 2B). At 48 h post‐fertilization (hpf), the proportion of embryos developing to the 4‐cell stage was significantly delayed in the PARP7‐KD group (PARP7‐KD vs. control: ∼10% vs. ∼90%). Subsequently, while a proportion of the developmentally delayed 2‐cell embryos in the PARP7‐KD group progressed through the 4‐cell and morula stages, the final blastocyst rate was only 15%, significantly lower than the 80% rate observed in the control group (Figure 2C,D).

To determine the ultimate fate of these delayed 2‐cell embryos while minimizing positional disturbance during group culture, we performed real‐time observation using time‐lapse imaging of individual embryos. Confirming the previous observation, PARP7‐KD embryos predominantly remained arrested or exhibited severe delay at the 2‐cell stage at 48 hpf. At 60 hpf, while control embryos had formed a morula, PARP7‐KD embryos were mostly at the 4‐cell stage or had begun to fragment. Culturing individual 2‐cell embryos further demonstrated that PARP7‐KD resulted in significant 2‐cell arrest/delay​ and a substantially reduced blastocyst rate (Figure 2E,F).

Given that minor ZGA occurs in zygotes and early 2‐cell embryos, while major ZGA takes place during the mid‐to‐late 2‐cell stage [1, 2], we assessed the impact of this developmental arrest on ZGA by examining representative genes. mRNA levels of minor ZGA genes, such as *Bex6*, *Ddit4l*, and *Mt2*, were reduced. Similarly, major ZGA genes, including *Dppa2*, *Dppa4*, *Gata4*, *Zfp707*, and *Nr5a2*, showed insufficient expression (Figure 2G). POLR2A, the largest subunit of the core transcriptional machinery RNA polymerase II, and its phosphorylation dynamics are central to ZGA: POLR2A phosphorylation at Ser5 (POLR2A‐Ser5‐P) is associated with transcription pre‐configuration and initiation, while phosphorylation at Ser2 (POLR2A‐Ser2‐P) drives transcriptional elongation [21]. Immunofluorescence staining of 2‐cell embryos revealed a significant decrease in POLR2A‐Ser2‐P signal in the PARP7‐KD group, whereas POLR2A‐Ser5‐P levels showed no significant difference compared to controls (Figure 2H,I). These results indicate that PARP7 knockdown causes developmental delay at the 2‐cell stage and reduces blastocyst formation. PARP7 deficiency impedes the activation of ZGA during the 2‐cell stage.

### 2.3 Inhibition of PARP7's ADP‐Ribosyltransferase Activity Significantly Delays 2‐Cell Development and Reduces Blastocyst Rate

**FIGURE 3:**
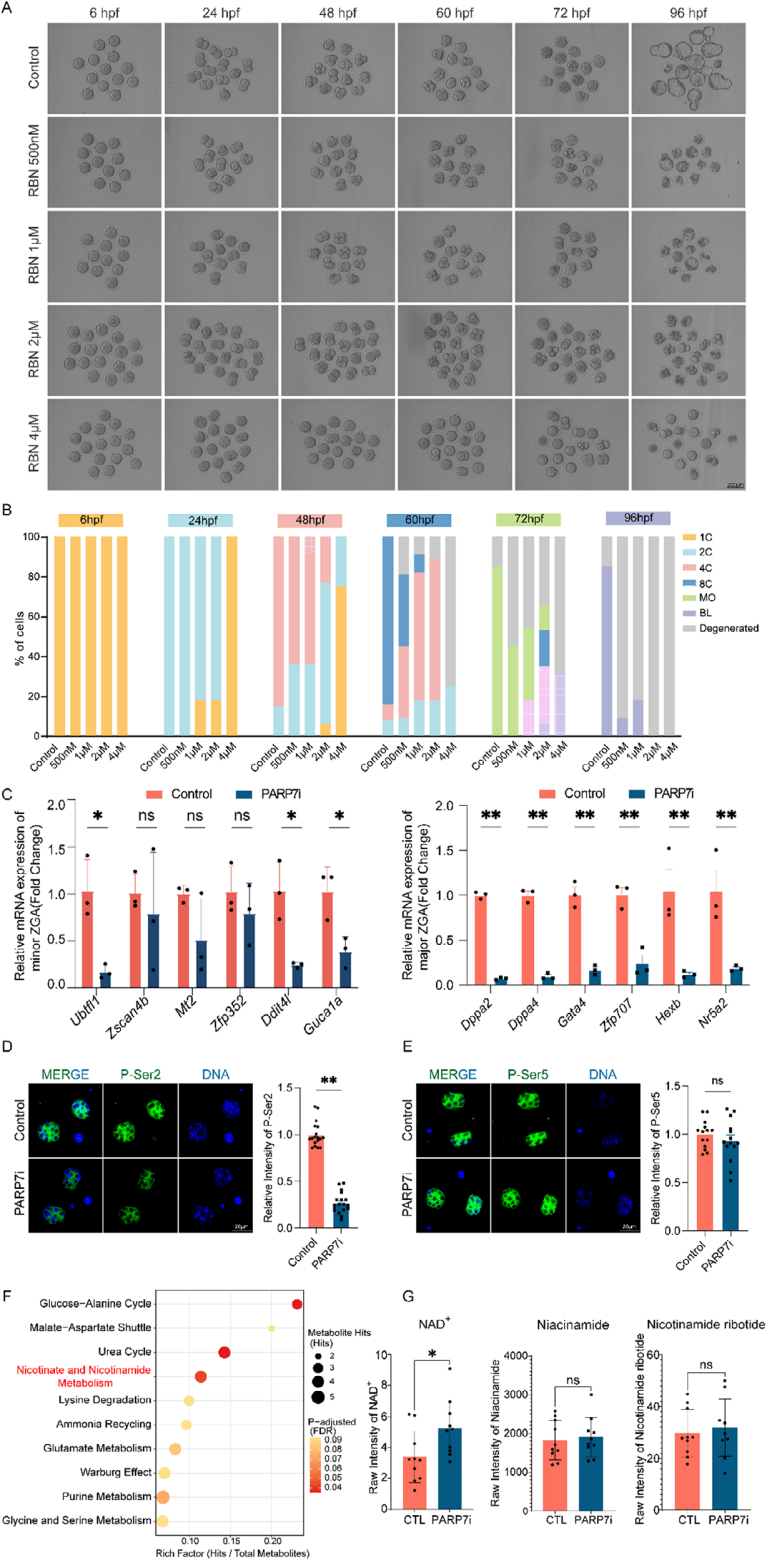
Inhibition of PARP7 catalytic activity significantly delays two‐cell development and reduces blastocyst rate. (A) Representative bright‐field images of embryos cultured from 6 h post‐fertilization (hpf) to 96 hpf in the presence of different concentrations of the PARP7 inhibitor RBN‐2397 (RBN). (B) Stacked bar charts showing the percentage of embryos at each developmental stage at indicated time points, treated with specified concentrations of RBN (Control, 500 nm, 1 µm, 2 µm, and 4 µm, *N* > 60 per group from three independent experiments). (C) RT‐qPCR analysis of Minor ZGA genes (left) and Major ZGA genes (right) in 2‐cell embryos treated with RBN (PARP7i). PARP7 inhibition significantly downregulated the expression of ZGA markers. (D, E) Representative immunofluorescence images and quantitative analysis of RNA polymerase II C‐terminal domain serine 2 (P‐Ser2) phosphorylation (D) and serine 5 (P‐Ser5) phosphorylation (E) in 2‐cell embryos after RBN treatment. DNA was stained with DAPI (blue). Scale bar: 20 µm. (F) Metabolite Set Enrichment Analysis (MSEA) of differentially expressed metabolites in PARP7i‐treated embryos. The “Nicotinate and Nicotinamide Metabolism” pathway (highlighted in red) is significantly enriched. (G) Bar graphs showing the raw intensity of NAD, Niacinamide, and Nicotinamide ribotide in Control and PARP7i‐treated embryos. PARP7 inhibition leads to a significant accumulation of the substrate NAD^+^, confirming the blockade of NAD^+^ consumption. Statistical analysis was performed using Student's t‐test. ^**^ *p* < 0.01; ns indicates no significant difference.

As PARP7 is the most highly expressed mono‐ADP‐ribosyltransferase at the 2‐cell stage, we sought to determine the role of its catalytic activity in early development using its specific inhibitor, RBN‐2397. RBN‐2397 is a highly selective small‐molecule inhibitor that competitively binds to the NAD^+^‐binding domain of PARP7, thereby blocking its mono‐ADP‐ribosyltransferase (MART) activity and inhibiting target protein MARylation (mono‐ADP‐ribosylation) [22]. To establish a concentration‐dependent effect, we tested a gradient of RBN‐2397 concentrations (500 nm, 1 µm, 2 µm, 4 µm) on early embryonic development. While all concentrations caused varying degrees of 2‐cell developmental delay and reduced blastocyst rates, we selected 2 µm for subsequent experiments (PARP7‐inhibitor, PARP7i group) as it most closely phenocopied the effects of PARP7‐KD (Figure 3A,B).

To assess the impact on ZGA in these developmentally arrested 2‐cell embryos, we examined the expression of representative ZGA genes. mRNA levels of minor ZGA genes, such as *Ubtfl1*, *Ddit4l*, and *Guca1a*, were insufficient. Similarly, major ZGA genes, including *Dppa2*, *Dppa4*, *Gata4*, *Zfp707*, and *Nr5a2*, showed reduced expression (Figure 3C). POLR2A‐Ser2‐P and POLR2A‐Ser5‐P are important markers for assessing the transcriptional status of RNA Pol II. Immunofluorescence staining showed a significant decrease in POLR2A‐Ser2‐P, but not POLR2A‐Ser5‐P, in the PARP7i group (Figure 3D,E).

To determine the effect of PARP7 inhibition on NAD^+^ metabolism, we analyzed the metabolic profile of 2‐cell embryos. Indeed, the NAD^+^ metabolic pathway was disrupted in the PARP7i group. As expected from the inhibition of a primary NAD^+^‐consuming enzyme, the substrate NAD^+^ showed significant accumulation. In contrast, the levels of nicotinamide and nicotinamide riboside were not significantly altered (Figure 3F,G). Collectively, these results demonstrate that pharmacological inhibition of PARP7 impedes ZGA progression at the 2‐cell stage, leading to developmental delay and reduced blastocyst formation. Inhibiting PARP7's ADP‐ribosyltransferase activity causes dysregulation of NAD^+^ metabolism, indicating that PARP7's role in regulating early embryonic development depends on its catalytic activity.

### 2.4 PARP7 Deficiency Impairs the Activation of Both Minor and Major ZGA

**FIGURE 4:**
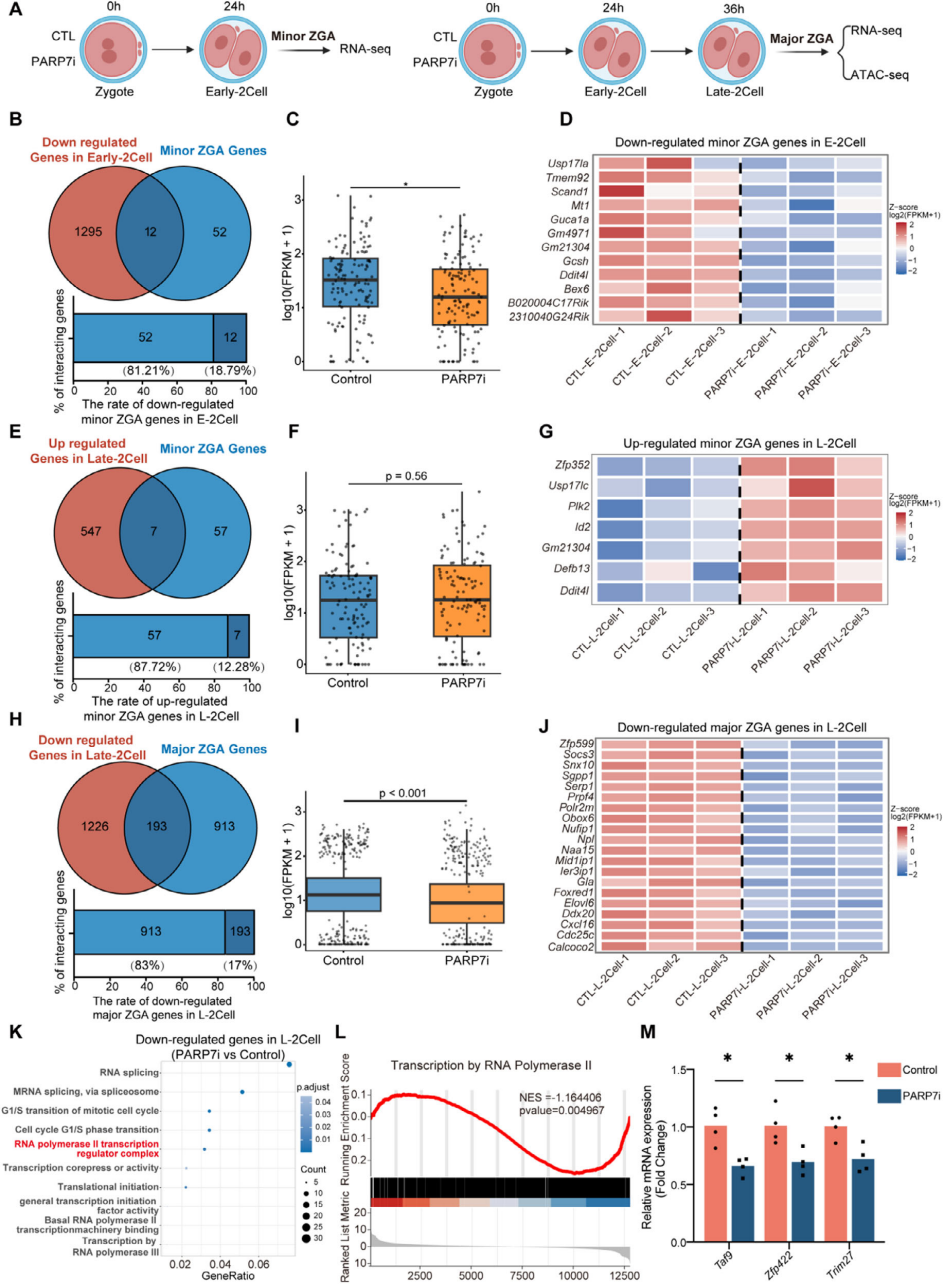
Loss of PARP7 function leads to failed activation of Minor ZGA and Major ZGA. (A) Schematic diagram of the RNA‐seq and ATAC‐seq experimental design. Zygotes were treated with a PARP7 inhibitor (PARP7i) or control (CTL), and samples were collected at the early 2‐cell stage (E‐2C, 24 hpf) for Minor ZGA analysis and at the late 2‐cell stage (L‐2C, 36 hpf) for Major ZGA analysis. (B) Venn diagram (top) showing the overlap between downregulated genes in PARP7i‐treated E‐2C embryos and the defined Minor ZGA gene set. Bar chart (bottom) showing the percentage of minor ZGA genes overlapping with the downregulated genes. (C) Box plots show the expression levels (log_10_(FPKM+1)) of minor ZGA genes. Statistical significance between the Control and PARP7i groups was assessed using the two‐sided Wilcoxon rank‐sum test at the E‐2C stage. (D) Heatmap displaying representative minor ZGA genes that were significantly downregulated upon PARP7 inhibition. These genes were identified through differential expression analysis (DESeq2) with the thresholds: padj < 0.05 and log2 fold change < −1. (E‐G) Analysis of minor ZGA genes at the late 2‐cell stage. (E) Venn diagram showing the intersection between upregulated genes at the late 2‐cell stage and the minor ZGA gene set. Bar chart (bottom) showing the percentage of minor ZGA genes overlapping with the upregulated genes. (F) Box plots comparing the expression levels (log_10_(FPKM+1)) of minor ZGA genes between control and PARP7i groups at the L‐2C stage. Statistical significance was assessed using the two‐sided Wilcoxon rank‐sum test. (G) Heatmap showing minor ZGA genes that remained abnormally upregulated or failed to be properly regulated at the late 2‐cell stage. These genes were identified through differential expression analysis (DESeq2) with the thresholds: padj < 0.05 and log2 fold change < −1. (H–J) Analysis of major ZGA genes at the late 2‐cell stage. (H) Venn diagram showing that 177 major ZGA genes were significantly downregulated in the PARP7i group. Bar chart (bottom) showing the percentage of major ZGA genes overlapping with the downregulated genes. (I) Box plots comparing the expression levels (log_10_(FPKM+1)) of major ZGA genes between control and PARP7i groups at the L‐2C stage. Statistical significance was assessed using the two‐sided Wilcoxon rank‐sum test. (J) Heatmap of the Major ZGA genes most significantly affected by PARP7i. These genes were identified through differential expression analysis (DESeq2) with the thresholds: padj < 0.05 and log2 fold change < −1. (K, L) Functional enrichment analysis of genes downregulated by PARP7i. GO enrichment bubble plot (K) and GSEA plot (L) show significant enrichment in RNA polymerase II transcription and RNA splicing pathways. (M) RT‐qPCR validation of the expression of representative major ZGA‐related genes (*Taf9*, *Zfp422*, *Trim27*) in PARP7i‐treated vs. control 2‐cell embryos. Data are presented as mean ± SEM. *padj < 0.05 (Student's t‐test). The central line in the box plots represents the median.

To further delineate the impact of PARP7 loss‐of‐function on ZGA progression during the 2‐cell stage, we separately analyzed transcriptomic changes in Early‐2Cell (minor ZGA phase) and Late‐2Cell (major ZGA phase) embryos (Figure 4A). During early embryonic development, zygotic genome activation (ZGA) occurs in two major phases: a set of so‐called “minor ZGA genes” are first activated in the zygote and early 2‐cell stage; subsequently, “major ZGA genes” initiate robust expression during the late 2‐cell stage, orchestrating subsequent developmental programs [23, 24]. First, to determine if PARP7 deficiency affects minor ZGA activation, we analyzed the expression levels of minor ZGA genes in Early‐2Cell embryos. In the PARP7i group, 18.79% of minor ZGA genes showed insufficient expression, exhibiting a downward trend (Figure 4B). Furthermore, the overall FPKM values for the minor ZGA gene set were decreased. Representative minor ZGA genes, such as *Usp17la*, *Tmem92*, and *Mt1*, were not significantly activated (the two‐sided Wilcoxon rank‐sum test, Figure 4C,D).

Second, to investigate whether PARP7 deficiency affects the timely silencing of minor ZGA, we analyzed minor ZGA gene expression in Late‐2Cell embryos. By intersecting upregulated differentially expressed genes in PARP7i late‐2Cell embryos with the minor ZGA gene set, we found that 12.28% of minor ZGA genes failed to be properly silenced and remained aberrantly upregulated (Figure 4E). While the overall minor ZGA gene set in PARP7i late‐2Cell embryos showed no significant difference, representative genes such as *Zfp352*, *Usp17lc*, and *Plk2* exhibited delayed silencing (the two‐sided Wilcoxon rank‐sum test, Figure 4F,G).

Next, to assess the effect of PARP7 deficiency on major ZGA activation, we analyzed major ZGA gene expression in Late‐2Cell embryos. In PARP7i late‐2Cell embryos, 193 major ZGA genes (17% of the set) showed insufficient expression, displaying a decreasing trend (Figure 4H). Consistently, the overall FPKM values for the major ZGA gene set were reduced. Representative major ZGA genes, including *Zfp599*, *Socs3*, and *Snx10*, were not robustly activated (the two‐sided Wilcoxon rank‐sum test, Figure 4I,J). Genes downregulated in the PARP7i group were significantly enriched in biological processes such as “RNA splicing,” “RNA polymerase II transcription regulator complex,” and “RNA polymerase II transcription machinery binding” (Figure 4K). Moreover, Gene Set Enrichment Analysis (GSEA) revealed a significant negative enrichment score for pathways related to RNA Pol II‐mediated transcriptional regulation (Figure 4L), indicating that PARP7 deficiency impairs this process. This was further validated by qPCR, showing significant downregulation of key RNA Pol II transcription‐related genes, including *Taf9*, *Zfp422*, and *Trim27*, in PARP7i Late‐2Cell embryos (Figure 4M). Integrated RNA‐seq analysis of Early‐2Cell and Late‐2Cell stages demonstrates that PARP7 deficiency causes failure in both minor and major ZGA activation and specifically disrupts RNA Pol II‐mediated transcriptional regulation.

### 2.5 PARP7 Deficiency Disrupts Chromatin Accessibility and Histone Reprogramming in Late 2‐Cell Embryos

**FIGURE 5:**
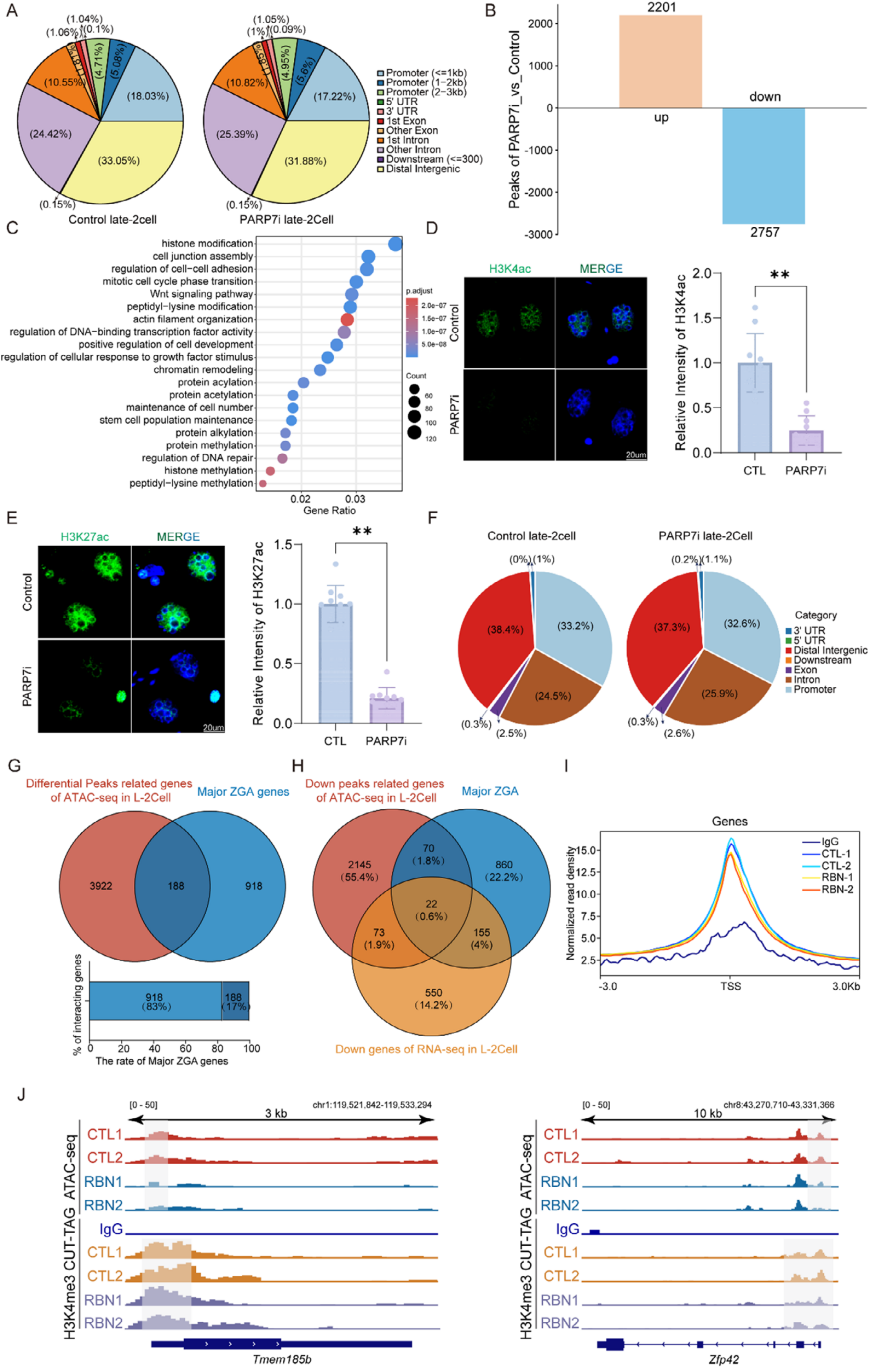
Loss of PARP7 function disrupts chromatin accessibility and histone reprogramming in late two‐cell embryos. (A) Genomic distribution of ATAC‐seq peaks in control and PARP7i‐treated late 2‐cell (L‐2C) embryos. (B) Bar chart showing the number of significantly differentially ATAC‐seq peaks (increased and decreased) in PARP7i‐treated embryos compared to controls. (C) Gene Ontology (GO) enrichment analysis of genes associated with differential ATAC‐seq peaks. Dot size represents the number of genes, and color represents significance (p‐adjust). (D, E) Immunofluorescence staining and quantitative analysis of H3K4ac (D) and H3K27ac (E) levels in control vs. PARP7i two‐cell embryos. Scale bar: 20 µm. (F) Pie charts showing the genomic distribution proportions of major ZGA gene categories in control and PARP7i groups (ATAC‐seq). (G) Venn diagram showing the overlap between major ZGA genes and genes associated with differential ATAC‐seq peaks in late two‐cell embryos. Bar chart (bottom) showing the percentage of major ZGA genes overlapping with the downregulated ATAC‐seq peaks‐related genes. (H) Ternary Venn diagram showing the intersection among downregulated ATAC‐seq peaks‐related genes, downregulated RNA‐seq genes, and major ZGA genes in the PARP7i group. (I) Metagene profile showing the average H3K4me3 signal intensity (CUT&Tag) around transcription start sites (TSS) in control and PARP7i‐treated embryos. CTL, Control. RBN‐1/2 represent biological replicates treated with the PARP7 inhibitor. (J) Representative genome browser tracks of ATAC‐seq and H3K4me3 CUT&Tag signals at the *Tmem185b* and *Zfp42* loci. Gray shaded areas highlight the reduction in chromatin openness and H3K4me3 enrichment at promoter regions in PARP7i‐treated embryos. Data are presented as mean ± SEM. ^**^ *p* < 0.01 (Student's t‐test).

During ZGA, the degree of chromatin openness determines the accessibility of regulatory elements (e.g., promoters, enhancers), directly influencing transcription factor binding and gene activation. To investigate the effect of PARP7 deficiency on chromatin openness, we performed ATAC‐seq. Compared to controls, the PARP7i group showed a reduced proportion (Promoter ≤ 1kb, 0.81%) of ATAC‐seq peaks localized to promoter regions (Figure 5A). PARP7 inhibition resulted in 2,201 significantly upregulated and 2,757 significantly downregulated peaks in Late‐2Cell embryos (Figure 5B; Figure S2A). GO analysis of genes associated with these differential peaks revealed significant changes in pathways including “histone modification,” “protein acylation,” and “chromatin remodeling” (Figure 5C). Under physiological conditions, H3K4ac and H3K27ac acetylation marks pre‐configure open chromatin to initiate major ZGA in Late‐2Cell embryos, serving as markers of enhanced transcriptional activity [25, 26]. PARP7 inhibition led to a significant decrease in the levels of both H3K4ac and H3K27ac in Late‐2Cell embryos (Figure 5D,E).

We next focused on changes specific to major ZGA genes. The proportion of ATAC‐seq peaks enriched at the promoters of major ZGA genes was reduced (Promoter ≤ 1kb, 0.6%) in the PARP7i group (Figure 5F). Intersection analysis identified 188 genes common between the differential ATAC‐seq peaks in Late‐2Cell embryos and the major ZGA gene set, representing 17% of major ZGA genes (Figure 5G). To pinpoint which major ZGA genes failed to initiate transcription, we intersected genes associated with downregulated ATAC‐seq peaks, downregulated transcripts from RNA‐seq (both in PARP7i vs. Control late‐2Cell), and the major ZGA gene set. This confirmed 22 major ZGA genes that were not properly transcriptionally activated (Figure 5H). Additionally, CUT&Tag for H3K4me3, a transcription activation marker at the Late‐2Cell stage, showed a decreasing trend in the enrichment of peaks at transcription start sites (TSS) in the PARP7i group (Figure 5I; Figure S3). Among these, representative major ZGA genes *Zfp42*and *Tmem185b*in the PARP7i group showed significant decreases in both ATAC‐seq peak signal and H3K4me3 enrichment at their promoters (Figure 5J). Combined analysis of ATAC‐seq and H3K4me3 CUT&Tag in Late‐2Cell embryos demonstrates that PARP7 deficiency disrupts chromatin accessibility and histone reprogramming.

### 2.6 Integrated PARP7 Interactome and Mono‐ADP‐Ribosylome Analyses Identify UHRF1 as a Target Regulating ZGA

**FIGURE 6:**
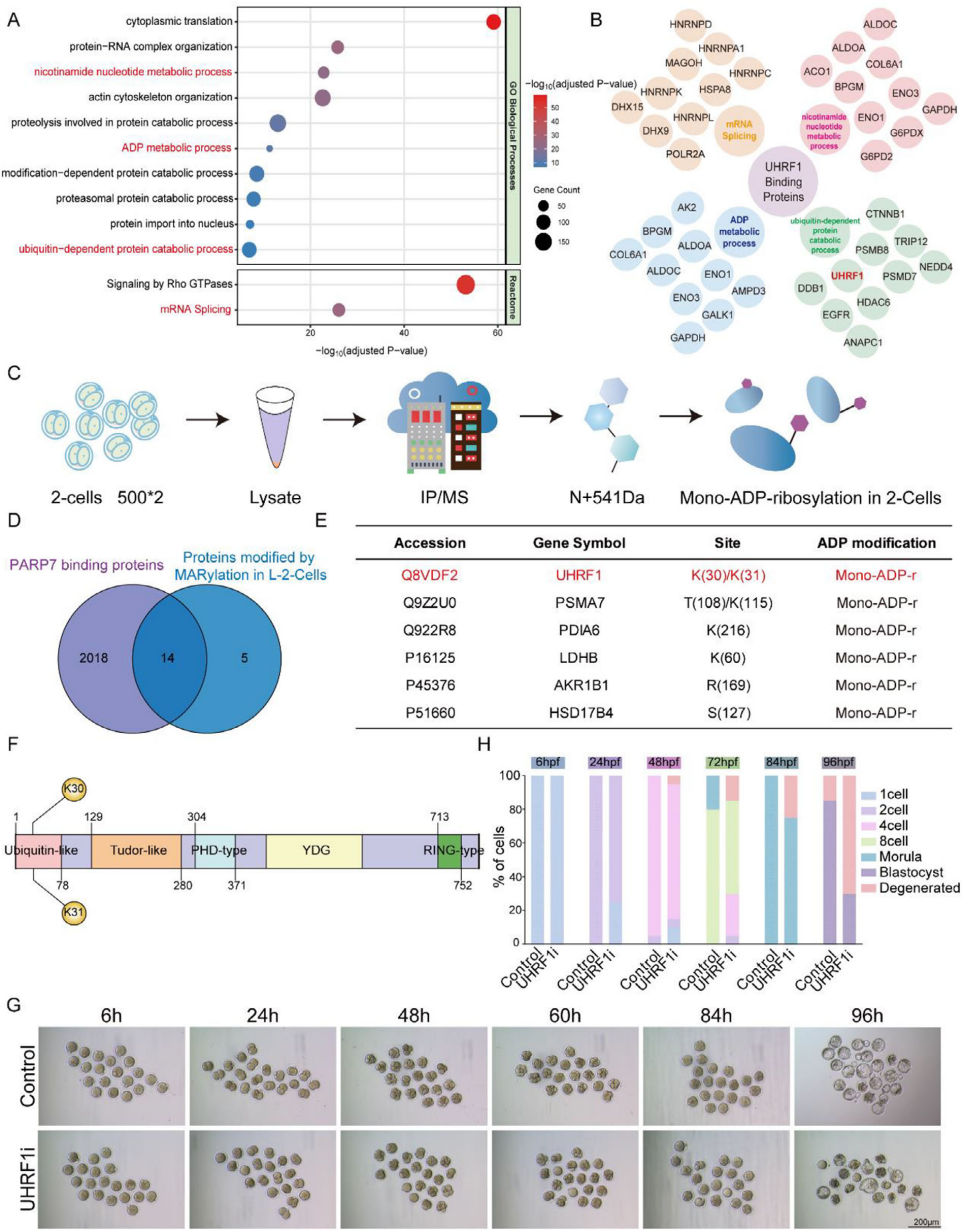
PARP7 Interactome and Mono‐ADP‐Ribosylome Jointly Reveal UHRF1 as a PARP7‐Regulated Target Protein in ZGA. (A) Bubble plots showing Gene Ontology (GO) biological processes (top) and Reactome pathways (bottom) enriched in the PARP7 interactome. Dot size represents the number of genes, and color indicates significance level (‐log_10_(P‐value)). (B) Protein‐protein interaction network diagram of the PARP7 interactome. (C) Schematic workflow of the experimental process for identifying mono‐ADP‐ribosylated proteins in two‐cell embryos using high‐resolution mass spectrometry. (D) Venn diagram showing the overlap between PARP7‐interacting proteins and candidate mono‐ADP‐ribosylated proteins. (E) Table listing core candidate proteins, with UHRF1 and its specific modification at K30/K31 sites highlighted. (F) Schematic diagram of the UHRF1 protein domain structure. (G) Representative bright‐field images of embryos treated with UHRF1 inhibitor (UHRF1i) compared to controls, cultured from 6 to 96 hpf. (H) Stacked bar chart quantifying the percentage of embryos at each developmental stage at specified time points following UHRF1 inhibition. N>60 per group from three independent experiments.

To identify the targets through which PARP7 regulates early embryonic development, we performed parallel PARP7 interactome analysis and mono‐ADP‐ribosylome profiling of 2‐cell embryos. The PARP7 interactome revealed affected pathways including “cytoplasmic translation,” “mRNA splicing,” and “ubiquitin‐dependent protein catabolic process.” Notably, pathways related to NAD^+^ metabolism, such as “nicotinamide nucleotide metabolic process” and “ADP metabolic process,” were also enriched, consistent with PARP7's function as an ADP‐ribosyltransferase (Figure 6A,B).

To precisely identify the sites of PARP7‐mediated mono‐ADP‐ribosylation (MARylation) on target proteins, we performed site‐specific ADP‐ribosylome analysis using 500 late 2‐cell embryos per biological replicate (*n* = 2). MARylation adds a mass shift of approximately 541 Da to the modified site. Our analysis identified 25 MARylated sites on 19 proteins in the 2‐cell embryo. Despite the technical limitations imposed by the low protein input from embryos and the sensitivity of the MARylome technique, 74% (14/19) of the MARylated proteins were also present in the PARP7 interactome. Strikingly, UHRF1 was found to harbor two adjacent MARylation sites at lysine residues K30 and K31 within its ubiquitin‐like domain (Figure 6C–F). UHRF1 has been previously implicated in regulating transcriptional regulation of the zygotic genome and DNA methylation in early embryos, though its upstream regulatory mechanisms remain unclear [27, 28]. Consistently, inhibition of UHRF1 in early embryos by our group also resulted in a significant decrease in blastocyst formation rate (Figure 6G,H). This integrated approach identifies UHRF1 as a key target protein through which PARP7 may regulate ZGA.

### 2.7 PARP7 Regulates UHRF1 Protein Stability, and UHRF1 Supplementation Partially Rescues the Transcriptional Defects Associated with ZGA

**FIGURE 7:**
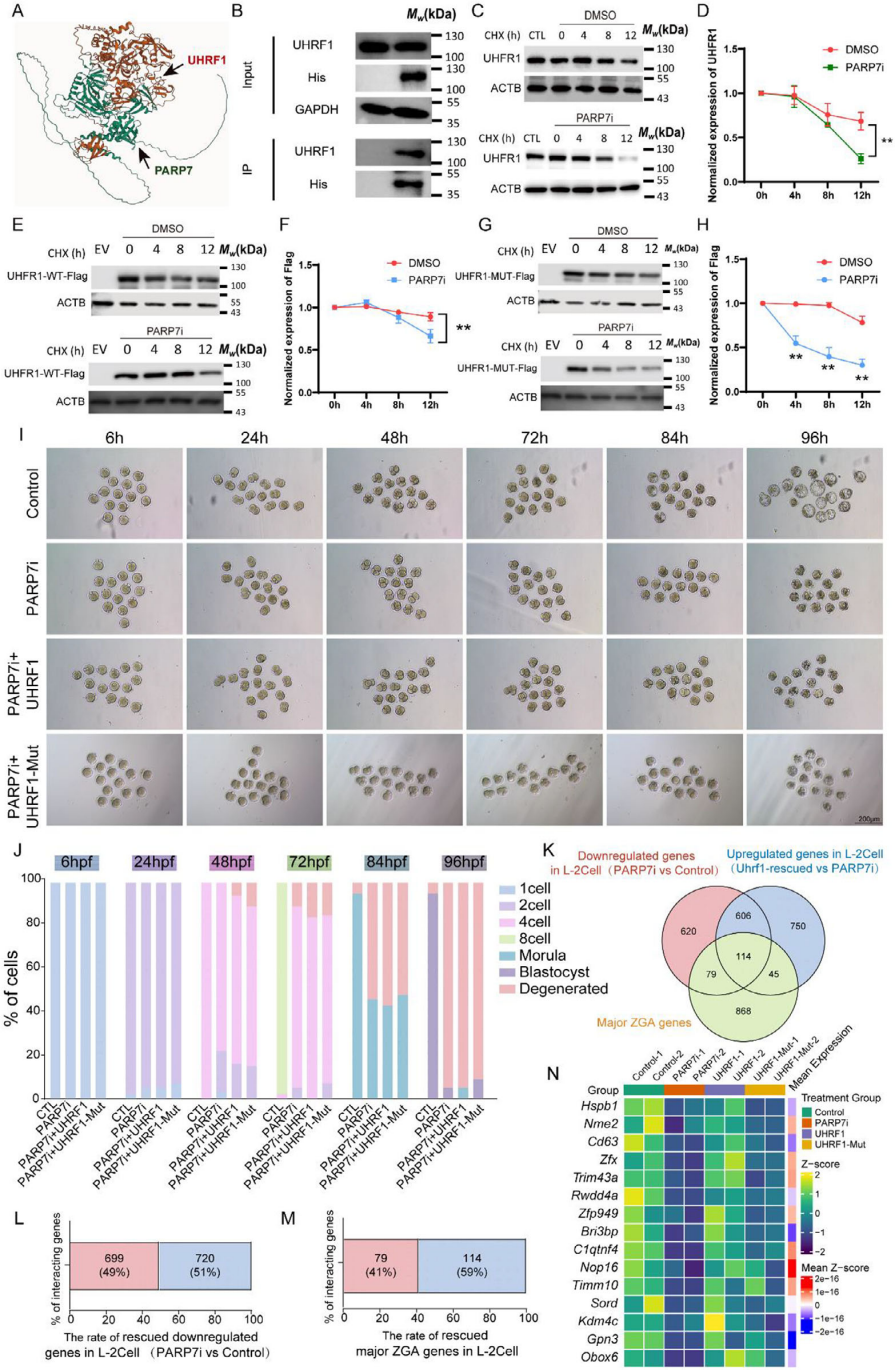
PARP7 regulates UHRF1 protein stability, and UHRF1 supplementation partially rescues the transcriptional defects associated with ZGA. (A) Structural modeling analysis predicts the interaction interface between PARP7 (green) and UHRF1 (red). (B) Co‐immunoprecipitation assay demonstrates the interaction between PARP7 and UHRF1. Cell lysates from HEK293T cells transfected with His‐tagged PARP7 were subjected to immunoprecipitation using an anti‐UHRF1 antibody. GAPDH serves as the loading control for Input. (C) CHX chase assay showing UHRF1 protein stability. Cells treated with DMSO or a PARP7 inhibitor were collected at indicated time points (0, 4, 8, 12 h) after CHX treatment. ACTB serves as the loading control. (D) Quantitative analysis of UHRF1 protein degradation kinetics determined from the CHX chase assay in (C). Data are normalized to ACTB levels and expressed relative to the 0 h time point (set as 1). (E) CHX chase assay showing the protein stability of Flag‐tagged wild‐type UHRF1 (UHRF1‐WT‐Flag). Cells transfected with UHRF1‐WT‐Flag and treated with DMSO or PARP7 inhibitor (PARP7i) were collected at indicated time points (0, 4, 8, 12 h) after CHX treatment. ACTB serves as the loading control. (F) Quantitative analysis of UHRF1‐WT‐Flag protein degradation kinetics determined from the CHX chase assay in (E). Data are normalized to ACTB levels and expressed relative to the 0 h time point (set as 1). (G) CHX chase assay showing the protein stability of Flag‐tagged mutant UHRF1 (UHRF1‐MUT‐Flag). Cells transfected with UHRF1‐MUT‐Flag and treated with DMSO or PARP7 inhibitor (PARP7i) were collected at indicated time points (0, 4, 8, 12 h) after CHX treatment. ACTB serves as the loading control. (H) Quantitative analysis of UHRF1‐MUT‐Flag protein degradation kinetics determined from the CHX chase assay in (G). Data are normalized to ACTB levels and expressed relative to the 0 h time point (set as 1). (I) Representative bright‐field images of mouse embryos from the control, PARP7i‐treated, and PARP7i + *Uhrf1* mRNA rescue groups, cultured from 6 hpf to 96 hpf. Overexpression of UHRF1 rescues the developmental arrest caused by PARP7 inhibition. Scale bar: 200 µm. (J) Stacked bar chart showing the percentage of embryos at each developmental stage for the indicated groups and time points. (K)Venn diagram showing the overlap among genes downregulated by PARP7i (red), genes upregulated by UHRF1 rescue (blue, Uhrf1‐rescued vs PARP7i), and defined Major ZGA genes (green) in late 2‐cell embryos. The intersection (114 genes) represents Major ZGA genes that are regulated by the PARP7‐UHRF1 axis. (L) Bar chart showing the percentage of PARP7i‐downregulated genes that are restored by UHRF1 overexpression. (M) Bar chart showing the percentage of PARP7i‐downregulated Major ZGA genes that are restored by UHRF1 overexpression. (N) Heatmap displaying the expression patterns (Z‐score) of representative rescued Major ZGA genes (from the intersection in j) across Control, PARP7i, and Rescue groups. Data are presented as mean ± SEM from three independent experiments. Statistical significance was analyzed using two‐way ANOVA. ^**^ *p* < 0.01; ns, not significant.

To elucidate how PARP7 regulates UHRF1, we first confirmed their physical interaction. Molecular docking using AlphaFold3 predicted a spatial interaction model between PARP7 and UHRF1 (Figure 7A). This interaction was further validated by co‐immunoprecipitation in 293T cells overexpressing PARP7‐His and UHRF1 (Figure 7B). We next asked whether PARP7 regulates UHRF1 protein stability. The cycloheximide (CHX) chase assay, a classical method to measure protein degradation rate and half‐life by inhibiting new protein synthesis [29], was employed. CHX treatment resulted in a gradual decrease in UHRF1 protein levels over time (Figure 7C,D).

We then investigated whether PARP7‐mediated stabilization of UHRF1 depends on the MARylation sites K30 and K31. We constructed UHRF1‐WT‐Flag and UHRF1‐Mutant‐Flag (K30A, K31A) plasmids. The CHX chase assay revealed that upon PARP7 inhibition, the protein level of UHRF1‐Mutant‐Flag rapidly decreased to approximately 39% and 29% at 8 and 12 h, respectively, whereas the UHRF1‐WT‐Flag protein maintained over 88% and 65% of its initial expression levels at the corresponding time points (Figure 7E–H).

Given that PARP7‐mediated MARylation stabilizes UHRF1, we tested whether supplementing wild‐type UHRF1 (UHRF1‐WT), but not the MARylation‐site mutant (UHRF1‐Mut; K30A, K31A), could ameliorate the ZGA defects caused by PARP7 deficiency. Despite failing to significantly improve the developmental progression or blastocyst rate of PARP7i‐treated embryos (Figure 7I,J), microinjection of *Uhrf1*‐WT mRNA, but not *Uhrf1*‐Mut mRNA, into PARP7i zygotes reversed the expression of relevant mRNAs at the molecular level (Figure 7I–K). RNA‐seq analysis of the three groups (Control, PARP7i, and PARP7i+UHRF1) revealed that 51% (720) of the downregulated transcripts were reversed in the UHRF1‐rescued group (Figure 7K,L). Moreover, 114 out of 193 downregulated major ZGA genes (59%) due to PARP7 inhibition were restored. Heatmap analysis demonstrated the specific rescue by UHRF1‐WT, but not by UHRF1‐Mut, of several representative major ZGA genes, such as *Hspb1*, *Cd63*, and *Zfx* (Figure 7M,N).

Collectively, these results demonstrate that PARP7 regulates UHRF1 protein stability via MARylation at K30/K31. UHRF1 overexpression partially rescued the transcriptional defects caused by PARP7 inhibition, as evidenced by the reversal of a subset of downregulated genes and major ZGA genes.

## 3 Discussion

**FIGURE 8:**
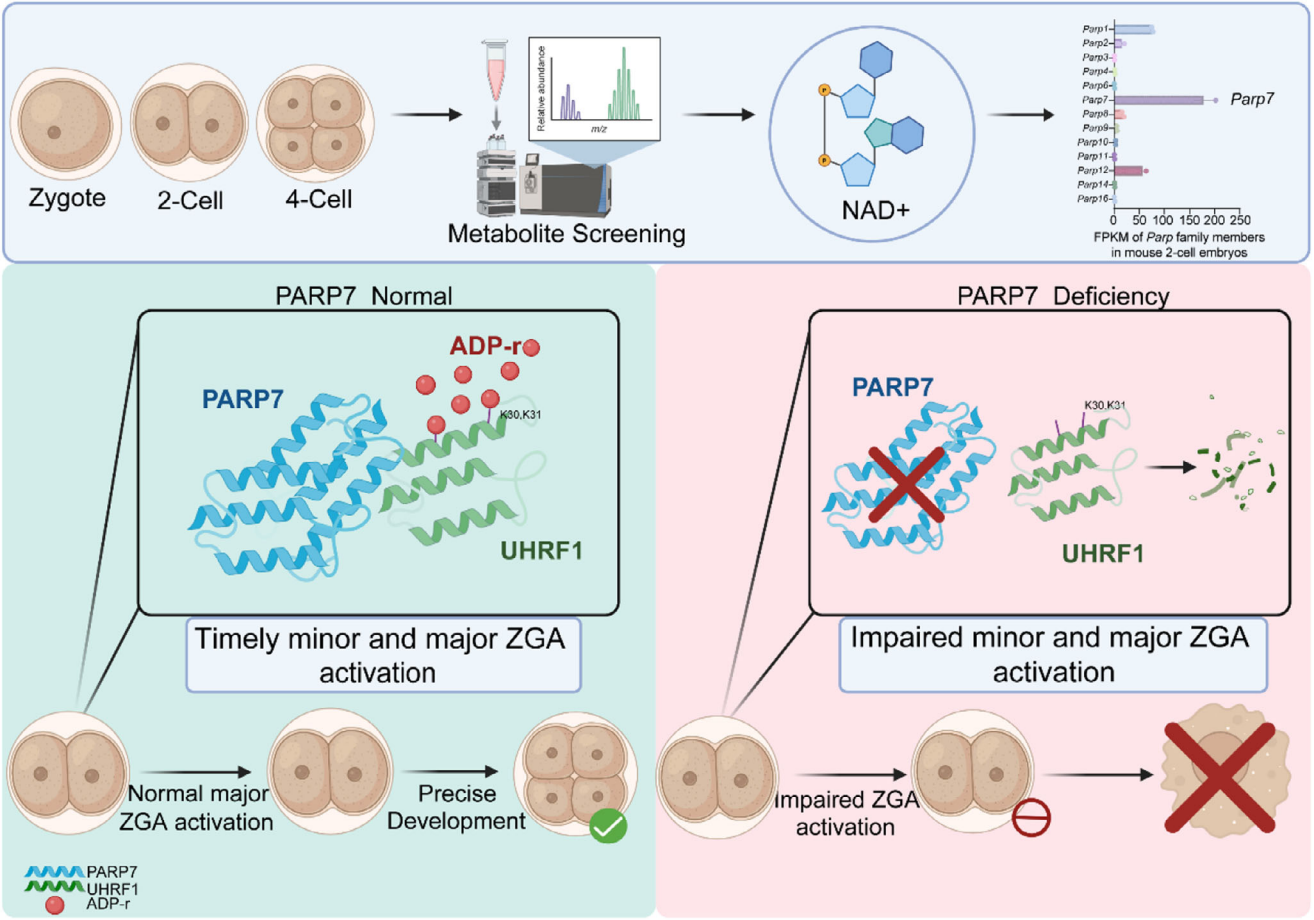
PARP7 Regulates the Activation of Minor ZGA and Major ZGA in Mouse Embryos by Influencing UHRF1 Protein Stability. Top Panel: Schematic diagram summarizing the research workflow of this study. Metabolomic screening of mouse preimplantation embryos (from zygote to 4‐cell stage) revealed a dynamic pattern of NAD^+^ consumption. Subsequent expression profiling identified PARP7 as a key NAD^+^ consumer specifically expressed at the 2‐cell stage. Bottom Left (Normal PARP7): Under physiological conditions, PARP7 utilizes NAD^+^ to catalyze the mono‐ADP‐ribosylation (ADP‐r) of UHRF1 at lysine residues 30 and 31 (K30, K31). This modification prevents the degradation of UHRF1, thereby stabilizing the protein and ensuring the timely initiation of both Minor and Major zygotic genome activation (ZGA), as well as precise embryonic development. Bottom Right (PARP7 Deficiency): In the absence of PARP7 (PARP7 Deficiency), UHRF1 fails to be mono‐ADP‐ribosylated. Consequently, the unmodified UHRF1 becomes unstable and undergoes rapid degradation. The loss of UHRF1 protein leads to impaired ZGA, ultimately causing developmental arrest at the 2‐cell stage.

This study establishes a novel metabolic‐epigenetic regulatory axis essential for licensing the embryonic genome. We demonstrate that a programmed fluctuation in a core metabolite, NAD^+^, is not a passive byproduct but an active developmental cue. This cue is sensed and transduced by the mono‐ADP‐ribosyltransferase PARP7, which translates the metabolic signal into a specific post‐translational instruction—the MARylation and stabilization of the epigenetic regulator UHRF1(Figure 8). This cascade ensures proper chromatin remodeling and RNA Polymerase II‐mediated transcriptional activation, thereby defining a complete regulatory circuit from metabolite to epigenetic modification to gene expression output during the critical 2‐cell stage. Our findings position metabolism as a primary conductor, rather than a secondary accompaniment, of the zygotic genome activation (ZGA) program.

Our work directly addresses the long‐standing question of how metabolism guides developmental transitions. While the importance of metabolites as co‐factors for epigenetic enzymes is recognized (e.g., acetyl‐CoA for HATs, α‐KG for KDMs), the role of NAD^+^ dynamics in preimplantation development has been speculative [5]. We provide definitive evidence that NAD^+^ levels undergo a precise, stage‐specific nadir concurrent with the onset of major ZGA. This positions NAD^+^ as a potential “metabolic timer.” The functional consequence is revealed by the concomitant, specific upregulation of its consumer, PARP7. Unlike the broadly studied sirtuins, whose protein levels did not correlate with the NAD^+^ dip [19], PARP7's expression is exquisitely timed. This suggests a model where the embryo utilizes a transient drop in a global metabolite to selectively activate a specific enzymatic “writer,” thereby initiating a dedicated downstream cascade. This mechanism elegantly explains how a ubiquitous metabolic shift can be channeled into a precise, stage‐appropriate developmental outcome, moving beyond correlative observations to establish causality [6].

The identification of PARP7 as the central effector in this pathway is a significant finding. While PARP1's role in embryo DNA damage response is known [30, 31, 32], PARP7's function in development was entirely unexplored. Its unique expression peak at the 2‐cell stage signifies a role specialized for ZGA, distinct from its functions in cancer or immunity [10, 12]. The profound developmental arrest upon its loss‐of‐function underscores its non‐redundancy. Crucially, we show that its catalytic activity is indispensable, as chemical inhibition with RBN‐2397 [22] phenocopied genetic knockdown. This establishes ADP‐ribosylation, specifically MARylation, as a critical regulatory modification during this developmental window, expanding its functional repertoire beyond DNA repair and stress response.

A key mechanistic breakthrough is the identification of UHRF1 as a functionally relevant substrate of PARP7. UHRF1 is a multifunctional epigenetic regulator with known roles in maintaining DNA methylation and heterochromatin structure [33, 34]. Our data reveal a novel regulatory layer: MARylation at K30/K31 modulates UHRF1 protein stability. The direct interaction between PARP7 and UHRF1, coupled with the instability of the MARylation‐site mutant, provides a direct molecular link. The partial but significant rescue of ZGA defects by UHRF1 overexpression firmly places it downstream of PARP7 in this pathway. This PARP7‐UHRF1 axis bridges the metabolic event to the chromatin landscape: UHRF1 stabilization facilitates the establishment of permissive histone marks (H3K4ac, H3K27ac) [25, 26] and chromatin accessibility, which in turn enables the recruitment and productive elongation of RNA Pol II (marked by POLR2A‐Ser2‐P) [21]. This connects NAD^+^ consumption directly to the epigenetic and transcriptional machinery.

It is noteworthy that although UHRF1 overexpression partially reversed the transcriptional consequences of PARP7 deficiency, its ability to rescue the embryonic developmental progression was limited (Figure 7I,J). This suggests that the MARylation network mediated by PARP7 may be more complex. While UHRF1 is a key MARylation substrate identified at the 2‐cell stage, the functional restoration of UHRF1 alone may be insufficient to compensate for all the downstream effects caused by PARP7 depletion. The incomplete phenotypic rescue could be collectively attributed to other, yet unidentified MARylated proteins, and/or the stringent dosage requirement of UHRF1 itself during development. Future studies are required to unveil the full repertoire of PARP7 substrates, thereby completing the mapping of its regulatory network controlling ZGA.

From a methodological perspective, this study exemplifies the power of applying cutting‐edge, low‐input multi‐omics technologies to the challenging context of mammalian embryos. The integration of ultra‐low input embryo metabolomics, CUT&Tag, ADP‐ribosylomics, and time‐lapse imaging has enabled the construction of a high‐resolution, causal map from metabolite to phenotype. The use of the highly selective inhibitor RBN‐2397 and the precise Trim‐away technique [20] provided robust tools for functional dissection in a system recalcitrant to conventional genetics, setting a precedent for future mechanistic studies in early development.

Several important questions emerge from our findings. First, what upstream signals trigger the specific transcriptional upregulation, such as PARP7 at the 2‐cell stage? Second, while we identified UHRF1, the full substrate landscape of PARP7 during ZGA likely includes other transcriptional and RNA processing factors, as hinted by our interactome data. Third, what is the precise physiological trigger for the NAD^+^ dip—is it driven by accelerated consumption (e.g., by other NAD^+^ consumers) or a regulated pause in synthesis? Finally, the partial nature of the UHRF1 rescue suggests that additional PARP7‐dependent pathways contribute to ZGA. Investigating these avenues will provide a more holistic understanding of this metabolic checkpoint.

## 4 Conclusions

In conclusion, we have delineated a coherent pathway wherein a metabolic oscillation (NAD^+^ depletion) activates a specific enzymatic sensor (PARP7) to modify and stabilize a key epigenetic regulator (UHRF1), thereby enabling the chromatin reprogramming necessary for ZGA. This work fundamentally advances our understanding of the metabolic control of embryogenesis, revealing MARylation as a crucial regulatory layer in early development. It positions the PARP7‐UHRF1 axis not only as a pivotal node in reproductive biology but also as a potential target for addressing metabolic‐linked embryo arrest, with implications for advancing assisted reproductive technologies and our basic knowledge of life's beginnings.

## 5 Experimental Section

### 5.1 Animals

B6D2F1 hybrid mice (8‐week‐old females and 12‐ to 16‐week‐old males) were purchased from Beijing Vital River Laboratory Animal Technology Co., Ltd. These mice were bred from C57BL/6 female and DBA/2 male parents. All animals were housed in a specific pathogen‐free (SPF) barrier facility at Nanjing Drum Tower Hospital Affiliated to Nanjing University Medical School, with environmental parameters maintained as follows: temperature 20°C–22°C, relative humidity 40%–70%, and a 12‐h light/dark cycle (07:00–19:00). Food and water were provided ad libitum. All experimental procedures were approved by the Animal Ethics Committee of Nanjing Drum Tower Hospital (Approval No. 2025AE01013).

### 5.2 In Vitro Fertilization and Embryo Culture

Oocytes were collected from 8‐week‐old female mice superovulated with 10 IU PMSG (Ningbo Sansheng Biotechnology), followed by 10 IU hCG (Ningbo Sansheng Biotechnology) 48 h later. At 14–16 h post‐hCG injection, mice were euthanized by cervical dislocation. Cumulus‐oocyte complexes were collected from the oviductal ampulla, and MII oocytes were selected and placed in pre‐equilibrated G‐IVF PLUS droplets (Vitrolife, #10136) under mineral oil. Sperm were collected from the cauda epididymis of male mice into G‐IVF PLUS medium, minced to release spermatozoa, and capacitated for 1 h at 37°C. For fertilization, capacitated sperm were added to oocyte‐containing droplets (sperm concentration was optimized to prevent polyspermy) and co‐incubated for 4–6 h. Subsequently, zygotes were washed and cultured in G‐1 PLUS droplets (Vitrolife, #10128) under mineral oil at 37°C in a 5% O_2_, 5% CO_2_, and 90% N_2_ atmosphere. Embryos were collected at specified hours post‐fertilization (hpf): zygote (6 hpf), 1‐cell (12 hpf), early 2‐cell (20 hpf), late 2‐cell (32 hpf), 4‐cell (45 hpf), 8‐cell (55 hpf), morula (72 hpf), and blastocyst (96 hpf).

### 5.3 Western Blot

Embryos (20–30 per sample) were washed and lysed in PBS‐PVA containing an equal volume of 2× Laemmli buffer (Bio‐Rad, #L00426C) with β‐mercaptoethanol. Lysates were denatured at 105°C for 10 min, briefly centrifuged, and separated on 10% SDS‐PAGE gels. Proteins were transferred to PVDF membranes (Millipore, #03010040001) in chilled transfer buffer at 90 V for 1.5–2 h at 4°C. Membranes were blocked with 5% non‐fat milk in TBST for 1 h, incubated overnight at 4°C with primary antibody (e.g., PARP7, Abcam ab200390, 1:500), and then with HRP‐conjugated secondary antibody (goat anti‐rabbit IgG, ZSGB‐Bio #ZB‐2301, 1:10,000) for 1 h at room temperature. Signals were developed with Immobilon ECL substrate (Millipore, #WBKLSO500) and analyzed using ImageJ and GraphPad Prism 10.0. Antibody details are provided in Table S1.

### 5.4 Immunofluorescence Staining

Embryos were fixed in 4% paraformaldehyde (PFA; Sigma, #158127) for 30 min at room temperature. Following PBS washes, samples were permeabilized with 0.5% Triton X‐100 (Sangon, #A110694) for 20 min. Blocking was performed with 5% fetal bovine serum (FBS; Gibco, #1645615) in PBS for 1 h. Embryos were incubated with primary antibody (e.g., PARP7, Invitrogen PA5‐98589, 1:200) overnight at 4°C, washed with PBST, and then incubated with fluorescent secondary antibody (Alexa Fluor 488 goat anti‐rabbit IgG, Invitrogen A‐11008, 1:1000) for 1 h in the dark. After counterstaining with DAPI, samples were mounted on glass slides with ProLong Gold Antifade Mountant (Invitrogen, #P36931) and imaged using a LEICA TCS‐SP8 confocal microscope. Fluorescence intensity was quantified with ImageJ.

### 5.5 Quantitative PCR (qPCR)

For each developmental stage, ten embryos were washed in PBS‐PVA, transferred to 0.2 mL tubes, flash‐frozen in liquid nitrogen, and stored at −80°C. cDNA was amplified from embryos using the Single Cell Sequence Specific Amplification Kit (Vazyme, #P621) with a 0.1 µm primer pool per target. The 3.1 µL reaction contained 2.5 µL 2× Reaction Mix, 0.5 µL primer pool, and 0.1 µL RT/Taq enzyme. After freezing at −70°C for 2 min and centrifugation, PCR was run as follows: 50°C for 60 min; 95°C for 3 min; 17 cycles of 95°C for 15 s and 60°C for 15 min. Amplified products were diluted and used for qPCR with ChamQ SYBR Master Mix (Vazyme, #Q32‐02) in a 10 µL system. The cycling conditions were: 95°C for 3 min; 40 cycles of 95°C for 15 s, 60°C for 30 s, and 72°C for 30 s; and a final extension at 72°C for 1 min. Relative expression was calculated by the 2^(‐ΔΔCt) method using 18S rRNA as the internal control. Data were analyzed with GraphPad Prism 10.0. Primer sequences are listed in Table S2.

### 5.6 PARP7 Knockdown via Trim‐Away

The mCherry‐Trim21 plasmid was linearized with NotI (NEB), followed by phenol‐chloroform extraction and ethanol precipitation. mRNA was synthesized using the mMESSAGE mMACHINE T7 Transcription Kit (Invitrogen AM1344), polyadenylated with the Poly(A) Tailing Kit (Invitrogen AM1350), and purified with the MEGAclear Kit. Purity (A260/A280 > 1.8) and integrity were verified by Nanodrop and agarose gel electrophoresis, respectively. The pcDNA3.1(+)‐N‐DYK‐*Uhrf1* and pcDNA3.1(+)‐N‐DYK‐K30A‐K31A‐*Uhrf1* plasmids were synthesized by GenScript (Nanjing, China).

A mixture containing anti‐PARP7 antibody (Abcam ab200390, 1 µg/µL) alone (control) or combined with mCherry‐Trim21 mRNA (200 ng/µL) (PARP7‐KD group) was prepared and stored at 4°C. Approximately 10 pL of the mixture was injected into the cytoplasm of PN‐stage zygotes using a FemtoJet microinjector (Eppendorf). Embryos were cultured in G‐1 PLUS medium for 24 h. Knockdown efficiency was evaluated by mCherry‐Trim21 fluorescence under a fluorescence microscope (Nikon).

### 5.7 Embryo Transcriptome Sequencing (RNA‐Seq)

Zonae pellucidae were removed with acidic Tyrode's solution. Embryos were washed in PBS, transferred to 0.2 mL tubes containing 1 µL Sample Buffer, mixed with 1.5 µL nuclease‐free water, and stored at −80°C. cDNA was synthesized and amplified using the Single Cell Full‐Length mRNA‐Amplification Kit (Vazyme N712), purified with VAHTS DNA Clean Beads, and quantified with Qubit. Libraries were prepared with the TruePrep DNA Library Prep Kit (Vazyme TD501) and indexed with the TruePrep Index Kit (Vazyme TD202). Sequencing was performed on an Illumina NovaSeq platform. Differentially expressed genes (padj ≤ 0.05, log_2_|fold change| ≥ 1) were identified using DESeq2, followed by GO enrichment and GSEA analysis.

### 5.8 Co‐Immunoprecipitation‐Mass Spectrometry (IP‐MS) and Co‐IP

Cells were washed with PBS and lysed in RIPA buffer (150 mm NaCl, 1% NP‐40, 50 mm Tris pH 8.0, 1 mm EDTA) containing protease inhibitors (Roche, #4693132001). Lysates were scraped, sheared with a syringe, sonicated (100 W, 2 s on/2 s off, 1 min), and incubated at 4°C for 30 min. Supernatants were collected after centrifugation at 15 000 rpm for 5 min. Protein concentration was determined with a BCA assay (Thermo, 71285‐3). For IP, 150 µg of protein was reserved as input, and the remainder was pre‐cleared with Protein A/G Agarose (Abmart, A0001M) for 2 h at 4°C. Pre‐cleared lysates were incubated with His‐beads (Thermo, 88221) overnight at 4°C. Beads were washed with RIPA buffer, and bound proteins were eluted with 2× SDS loading buffer for Western blot or MS analysis. For Co‐IP validation, 293T cells were transfected with the His‐PARP7 plasmid. Lysates were subjected to IP with His‐beads, and co‐precipitated POLR2A was detected by Western blot (Proteintech, 20655‐1‐AP, 1:500).

### 5.9 Micro‐Embryo Metabolomics

Embryos (10 embryos as a pool) were washed three times in 50 µL droplets of 140 mm ammonium formate aqueous solution to remove residual culture medium and salts from the surface, thereby minimizing interference with downstream mass spectrometry signals. Individual pools were then carefully transferred to PCR tubes containing 15 µL of extraction solution (50% methanol in water, v/v, with 1% formic acid, v/v). Cell lysis was performed by repeated pipetting to generate fluid shear stress via the pipette tip, facilitating metabolite extraction.

The resulting embryo extracts were loaded onto a single‐cell mass spectrometer (SinCell‐100, China Innovation Instrument Co., Ltd., Ningbo, China) for metabolomic analysis. The system enables automated, high‐throughput single‐cell metabolomics, integrating a fully automated single‐cell sample preparation module for precise sub‐nanoliter liquid handling and ambient ionization, and a triple quadrupole mass analyzer for spectral acquisition [35, 36, 37]. Optimized parameters were as follows: positive ion mode, spray voltage = +2 kV, multiple reaction monitoring (MRM) mode, precursor ion mass range = *m/z* 50–1000, dwell time = 10 ms, fragmentor voltage = 166 V, collision energy = 30–40 eV.

Blank and embryo samples were acquired as raw mass spectrometry data files, and subsequently converted into an m × n matrix, where *m* and *n* denote the number of samples and metabolite features, respectively. After subtraction of baseline instrumental intensity, metabolites with signal‐to‐noise ratio (S/N) greater than 3 were retained as valid features for subsequent analysis [38, 39]. Specifically, the S/N value was calculated as the ratio of the intensity of the analyzed samples (embryos) to that of the blank samples (cell‐free extraction solvent). For comparative evaluation between groups, significant metabolites were selected using the following thresholds: *p* < 0.05, |log _2_(Fold change)| > 1.0 and VIP > 1.0. VIP values herein were derived from OPLSDA analysis performed with the R package ropls (version 11.34.0).

For clustering visualization of embryos across developmental stages, a UMAP analysis was conducted using the R package uwot (version 0.2.4) based on the shared significant metabolites among stages. Hierarchical clustering, heatmap, and volcano plot were plotted by https://www.bioinformatics.com.cn [40], an online platform for data analysis and visualization. KEGG pathway enrichment analysis for distinct metabolic clusters was performed using the MetaboAnalyst web server (version 6.0.0, https://www.metaboanalyst.ca), which operates via the underlying MetaboAnalystR package (version 4.0) [41, 42]. The corresponding bubble charts were subsequently visualized using the R package ggplot2 (version 3.5.2). The abundance trends and relative ratios of specific energy metabolites across developmental stages were statistically analyzed and plotted using GraphPad Prism (version 10.1.2).

### 5.10 ATAC‐Seq Assay

Late 2‐cell mouse embryos were selected as samples. Embryos were washed with cold PBS and permeabilized in lysis buffer containing 1% digitonin for 5 min on ice. Nuclei were pelleted after quenching with cold PBS. Library preparation was performed using the NovaPrep Hyperactive ATAC‐Seq Library Prep Kit (Vazyme, TD711). Nuclei pellets were resuspended in a 50 µL reaction mixture containing Tn5 transposase and 5× TTBL buffer, followed by tagmentation at 37°C for 30 min. Fragmented DNA was purified using ATAC DNA Extract Beads and amplified with TD711 primers. Amplified products were size‐selected with ATAC DNA Clean Beads. Libraries passing quality control (insert size > 200 bp) were sequenced on an Illumina NovaSeq platform (PE150). Raw reads were quality‐checked with FastQC, and adapters/low‐quality sequences were removed using fastp. Clean reads were aligned to the mm10 genome with Bowtie2. Open chromatin regions (peaks) were called using MACS2 and annotated with ChIPseeker. HOMER was used to analyze promoter regions (± 3 kb around TSS) and transcription factor motifs. Differential peaks were identified with DESeq2, and enriched GO terms/KEGG pathways were analyzed via clusterProfiler.

### 5.11 CUT&Tag Assay

Protein‐DNA interactions were assessed using the Hyperactive Universal CUT&Tag Assay Kit for Illumina Pro (Vazyme, TD904). Late 2‐cell embryos (*n* = 100) were bound to activated ConA Beads Pro and permeabilized with digitonin. Primary antibody (1 µL anti‐H3K4me3 or IgG control) and secondary antibody were sequentially incubated to guide pA/G‐Tnp Pro to target sites. Tagmentation was initiated in TTBL buffer, fragmenting DNA and adding adapters simultaneously. Fragments were extracted using DNA Extract Beads Pro after SDS‐mediated crosslink reversal. Libraries were amplified with dual‐index primers (TruePrep Index Kit V2, Vazyme TD202) and purified with VAHTS DNA Clean Beads. Sequencing data were processed with FastQC and fastp, aligned to the mouse genome using Bowtie2, and peaks were called with deepTools. GO enrichment of differential genes was performed with clusterProfiler (padjust < 0.05).

### 5.12 Statistical Analysis

All experiments were independently repeated ≥ 3 times. Measurement data are presented as mean ± SEM. GraphPad Prism 10.0 was used for statistical analysis: two‐group comparisons were analyzed by t‐test, and multi‐group comparisons by one‐way ANOVA. Furthermore, any analytical methods requiring specific description are detailed in the legends of the corresponding figures.

## Author Contributions

G.C., C.L., L.W., G.Y. and H.S. conceived and designed the research. G.C., A.C., Z.Z., Y.L., Y.L., R.S. and L.Y. performed the experiments. A.G. and G.L. analyzed the data. G.C., Y.M., G.Y. and H.S. offered the funding. G.C. wrote the manuscript.

## Ethics Statement and Animals

All experiments utilized ICR mice maintained in the specific pathogen‐free (SPF) facility at the Animal Experiment Center of Nanjing Drum Tower Hospital. All mice were maintained in a standard light/dark cycle with a regular diet and drinking water. All animal procedures were approved by the Institutional Animal Care and Use Committee (IACUC) of Nanjing Drum Tower Hospital (Approval No. 2025AE01013).

## Code Availability Statement

All custom code used for bioinformatics analysis in this study is available on GitHub at https://github.com/NathanST08/PARP7‐UHRF1‐ZGA.

## Conflicts of Interest

The authors declare no conflicts of interest.

## Supporting information




**Supporting File 1**: advs76136‐sup‐0001‐SuppMat.docx.

**Supporting File 2**: advs76136‐sup‐0002‐TableS1.xlsx.

**Supporting File 3**: advs76136‐sup‐0003‐TableS2.xlsx.

## Data Availability

All data supporting the findings of this study are available within the paper and its Supplementary information files. All the datasets used in this study are publicly available. The raw and processed data generated in this study, including RNA‐seq (CTL and PARP7i in Early and Late‐2Cell), ATAC‐seq (CTL and PARP7i in Late‐2Cell), and CUT&Tag (H3K4me3), have been deposited in the National Genomics Data Center, Beijing Institute of Genomics, Chinese Academy of Sciences, accession number PRJCA056092.
