# Supplementary material for: NAD+ Metabolism Licenses Zygotic Genome Activation via PARP7‐Mediated ADP‐Ribosylation of UHRF1 in Mouse Early Embryos
Source: Adv Sci (Weinh). 2026 Jun 15:e76136. Online ahead of print. doi: 10.1002/advs.76136 (PMC13336359; doi:10.1002/advs.76136)
Supplement: Supplementary file 1 — Supporting File 1: advs76136‐sup‐0001‐SuppMat.docx. [file ADVS-9999-e76136-s003.docx]

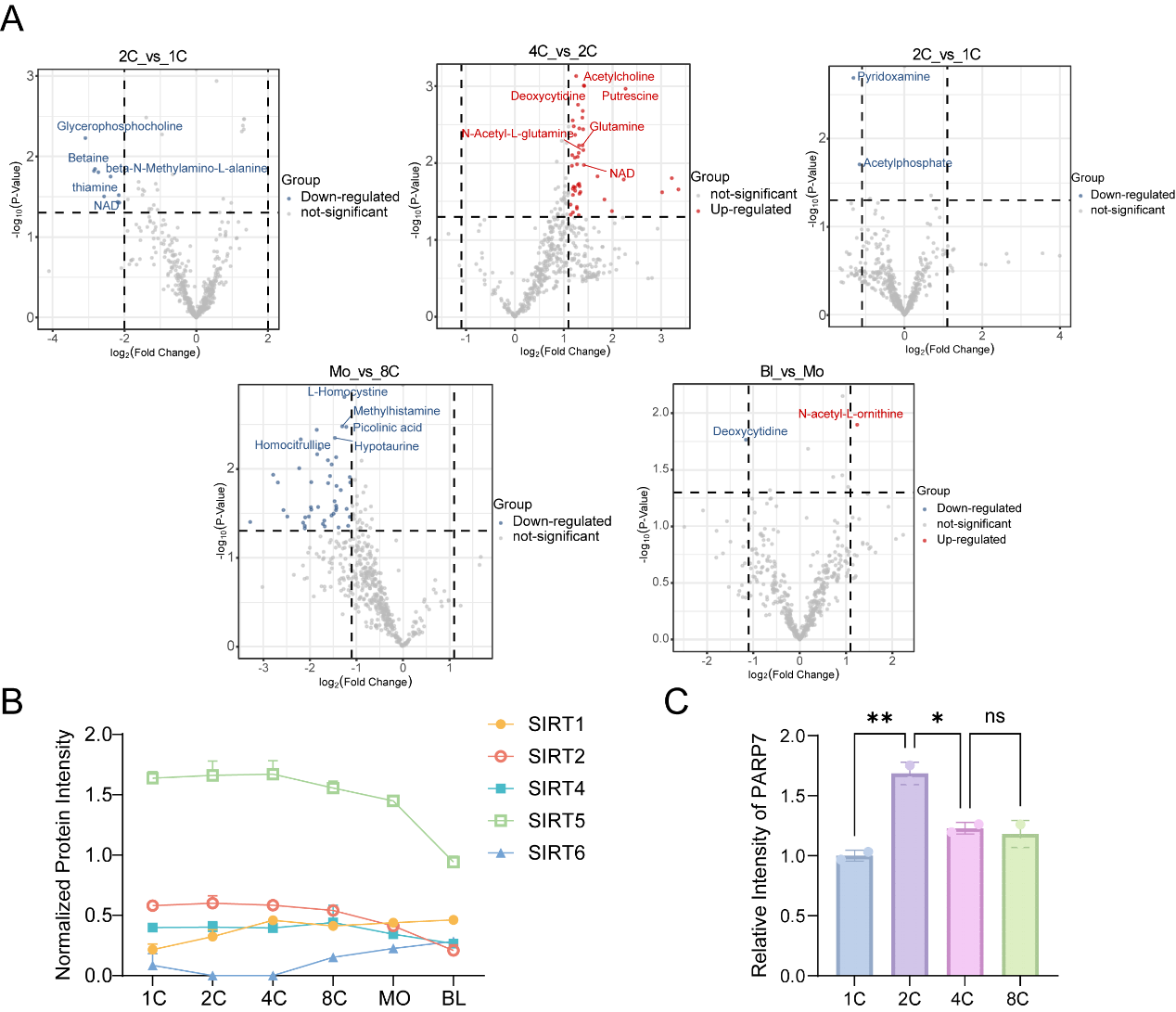


**Supplementary Figure S1. Differential Features of Metabolite Expression Across Mouse Developmental Stages from Zygote to Blastocyst.**

(A) Volcano plots displaying differentially expressed metabolites between specified developmental stages (2C vs. 1C, 4C vs. 2C, 8C vs. 4C, Mo vs. 8C, and Bl vs. Mo). Blue and red dots represent significantly downregulated and upregulated metabolites, respectively (|log₂FC| > 1, p < 0.05).

(B) Normalized protein intensity of Sirtuin family members (SIRT1, SIRT2, SIRT4, SIRT5, and SIRT6) during early embryonic development. Data are presented as mean ± SEM.

(C) Quantification of PARP7 protein expression from the zygote to the 8-cell stage.Data are presented as mean ± SEM. n=2 independent biological replicates. Statistical analysis was performed using Student’s t-test. *p < 0.05, **p < 0.01, ns indicates no significant difference.


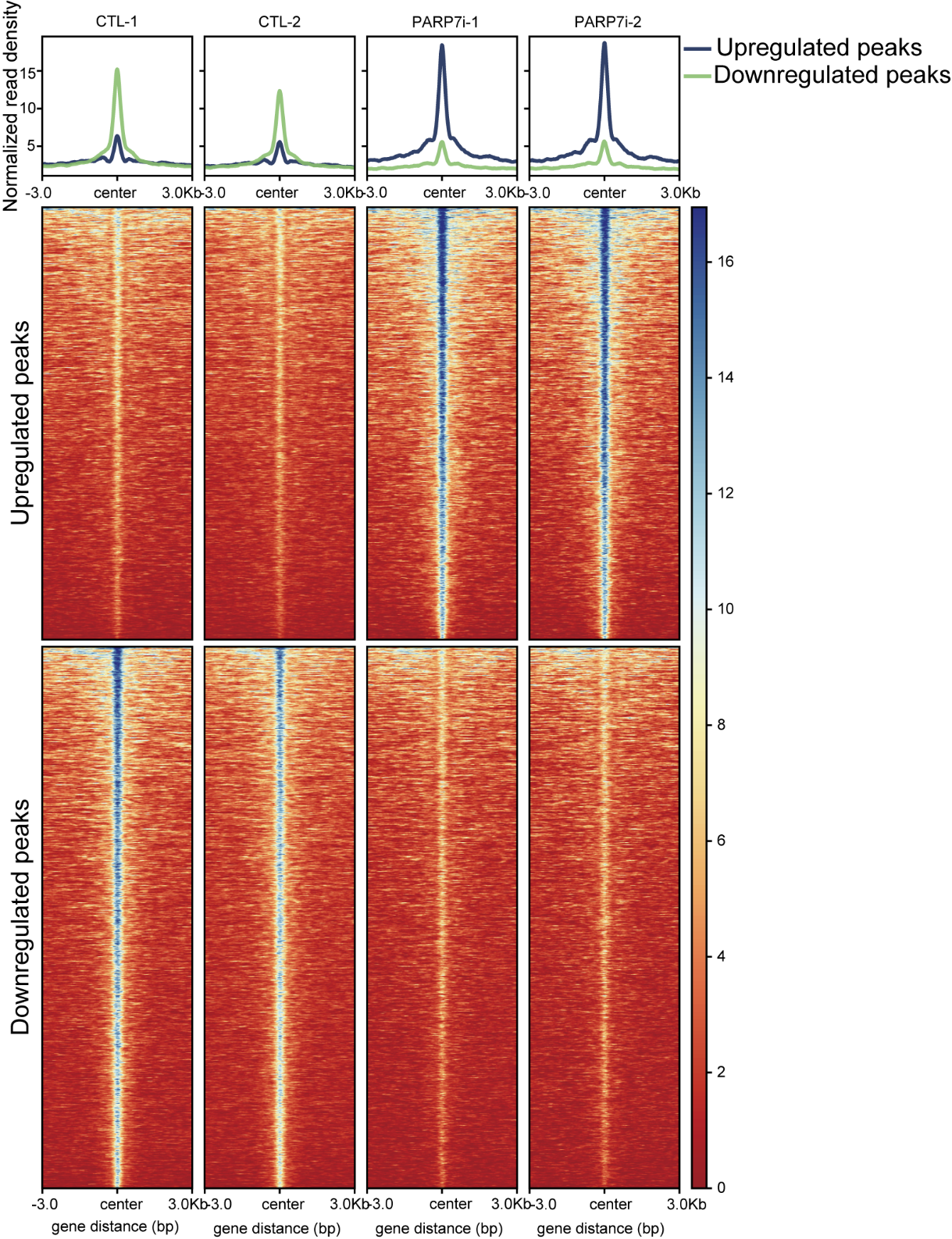


**Supplementary Figure S2. Loss of PARP7 function disrupts chromatin accessibility and histone reprogramming in late two-cell embryos.**

Read density profiles (top) and corresponding heatmaps (bottom) showing ATAC-seq signals centered on chromatin-accessible regions in control and PARP7 inhibitor-treated late two-cell embryos. Based on differential accessibility, signal intensity is categorized into "Upregulated peaks" and "Downregulated peaks" clusters. The color scale represents normalized read density from 0 to 16.


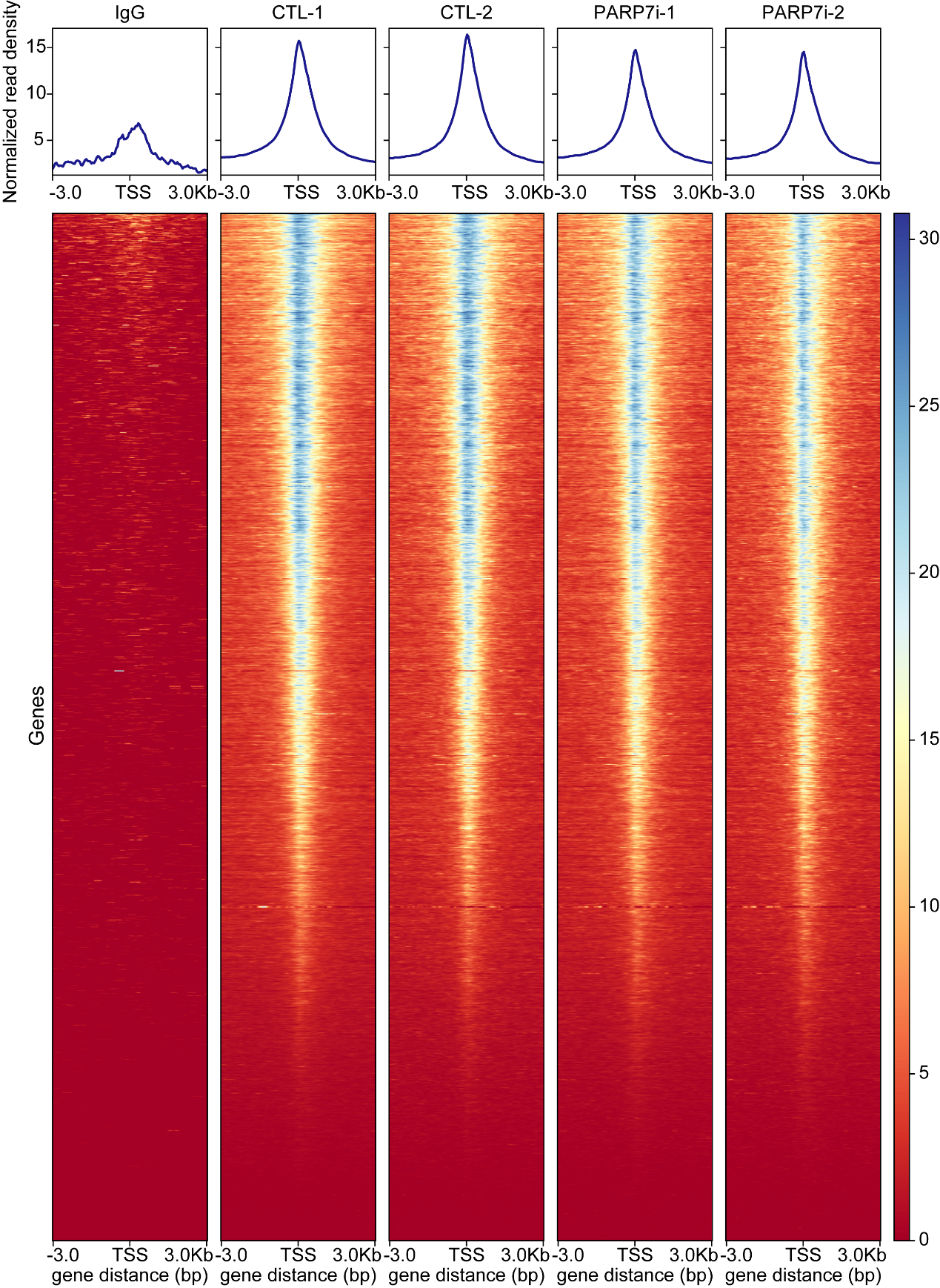


**Supplementary Figure S3. Global H3K4me3 profiles at TSS regions upon PARP7 inhibition.**

Heatmaps depicting normalized H3K4me3 CUT&Tag signal intensity (Z-score) across a 6-kb region (±3 kb) centered on the TSS of all RefSeq genes. Each row represents an individual gene, and genes are ranked by H3K4me3 signal intensity in the control group. Data are shown for control (left) and PARP7i-treated (right) embryos. The color scale indicates relative signal intensity from low (blue) to high (yellow). Average metagene profiles of H3K4me3 CUT&Tag signal at TSS regions (±3 kb). Signals for control and PARP7i groups are plotted. The y-axis represents the average normalized read density.

**Table S1. Antibody list used in this Study.**

**Table S2. Primer list used in this Study.**
